# Systematic characterization of cancer transcriptome at transcript resolution

**DOI:** 10.1038/s41467-022-34568-z

**Published:** 2022-11-10

**Authors:** Wei Hu, Yangjun Wu, Qili Shi, Jingni Wu, Deping Kong, Xiaohua Wu, Xianghuo He, Teng Liu, Shengli Li

**Affiliations:** 1 Precision Research Center for Refractory Diseases, Institute for Clinical Research, Shanghai General Hospital, Shanghai Jiao Tong University School of Medicine, Shanghai, 201620 China; 2 Department of Gynecological Oncology, Fudan University Shanghai Cancer Center, Shanghai, 200032 China; 3 Fudan University Shanghai Cancer Center and Institutes of Biomedical Sciences, Shanghai Medical College, Fudan University, Shanghai, 200032 China; 4 Institute of Big Data and Artificial Intelligence in Medicine, School of Electronics and Information Engineering, Taizhou University, Taizhou, 318000 China

**Keywords:** Cancer genomics, Data mining, Regulatory networks, Cancer therapy, Transcriptomics

## Abstract

Transcribed RNAs undergo various regulation and modification to become functional transcripts. Notably, cancer transcriptome has not been fully characterized at transcript resolution. Herein, we carry out a reference-based transcript assembly across >1000 cancer cell lines. We identify 498,255 transcripts, approximately half of which are unannotated. Unannotated transcripts are closely associated with cancer-related hallmarks and show clinical significance. We build a high-confidence RNA binding protein (RBP)-transcript regulatory network, wherein most RBPs tend to regulate transcripts involved in cell proliferation. We identify numerous transcripts that are highly associated with anti-cancer drug sensitivity. Furthermore, we establish RBP-transcript-drug axes, wherein PTBP1 is experimentally validated to affect the sensitivity to decitabine by regulating *KIAA1522-a6* transcript. Finally, we establish a user-friendly data portal to serve as a valuable resource for understanding cancer transcriptome diversity and its potential clinical utility at transcript level. Our study substantially extends cancer RNA repository and will facilitate anti-cancer drug discovery.

## Abstract (web summary)

Modification of transcribed mRNAs enables regulation of transcription but its extent in cancer cells is incompletely understood. Here, the authors analyse transcript assembly in over 1000 cancer cell lines and find unannotated transcripts are common, and are associated with drug sensitivity.

## Introduction

RNA transcripts, as the direct carriers of translational codes of proteins, are generated via diverse regulation and modification in particular contexts^1–3^. Under different contexts, transcriptional activity shows diversity in that different quantities of transcripts or totally distinct transcripts are produced from the same genes^4,5^. Transcriptional diversity greatly expands the encoding storage capacity of the genome for proteins in eukaryotes. Along with advancements in high-throughput RNA sequencing (RNA-seq)and the development of computational algorithms, transcriptional diversity has been largely brought to light in human diseases^6,7^. By reanalyzing RNA-seq data from 32 cancer types, Kahles et al. detected thousands of alternative splicing variants in many tumors^8^. Xiang et al. identified many alternative polyadenylation variants in 6398 tumor patients and 739 cancer cell lines, wherein 1971 variants were found to be clinically relevant^9^. These transcriptional variants generate specific RNA transcripts that may play important roles in tumor development. For example, a *LIN28B* variant, *LIN28B*-TST, was found to be specifically expressed in tumor samples and critical for cancer cell proliferation and tumorigenesis^10^. However, an integrative depiction of RNA transcripts in large-scale cancer transcriptomics data has been lacking. RNA-binding proteins (RBPs)have been shown to play crucial regulatory roles in post-transcriptional RNA expression, and aberrantly programmed RBP-RNA interactions modulate cancer initiation and progression^11–13^. Van Nostrand et al. reported the largest effort to date to systematically study the functions of 356 human RBPs. They combined the RBP binding data (eCLIP-seq)and RBP knockdown followed by RNA-seq data (KD-RNA-seq) to construct the RBP-gene regulation and RBP-splicing association. Furthermore, several RBPs have also been demonstrated to mediate the response of cancer cells to anti-cancer drugs, such as estrogen receptor α (ERα)^14^ and eukaryotic translation initiation factor 2 subunit beta (EIF2S2)^15^. Nevertheless, RBP regulation at the transcript level and its roles in mediating the response to anti-cancer drugs of cancer cells remain incompletely understood.

Human cancer-derived cell lines have been widely used as preclinical cancer models in cancer biology research and anti-cancer drug discovery^16^. Massive efforts have been made to delineate the molecular characteristics across large-scale cancer cell lines^17^. The Cancer Cell Line Encyclopedia (CCLE) project generated high-throughput sequencing data of hundreds of cancer cell lines at various molecular levels, including genomics, transcriptomics, epigenomics, proteomics, and metabolomics^18,19^. To further expedite drug discovery, the Genomics of Drug Sensitivity in Cancer (GDSC) project provides an unprecedented resource about drug sensitivity for 266 anti-cancer agents across 1,065 different cancer cell lines^20^, while the Cancer Therapeutics Response Portal (CTRP) provides information on responses to 481 compounds in 860 cancer cell lines^21^. Large biological troves and potential clinical applications have been discovered through the multifarious integrative characterization of sophisticated molecular landscapes across cancer cell lines^17,22,23^.

To comprehensively delineate the transcript atlas in cancer, we carried out a reference-based transcript assembly with RNA-seq data across more than 1000 cancer cell lines. The unannotated transcript *AC092803.3-u1* from the *AC092803.3* gene was experimentally validated and showed a higher expression level and clinical significance in multiple tumor types than the other transcript, *AC092803.3-a1*. Furthermore, RBP-transcript regulation and transcript-drug associations in cancer were combined to build RBP-transcript-drug axes, wherein PTBP1 was experimentally validated to affect the sensitivity to decitabine by regulating the expression of the *KIAA1522-a6* transcript of the *KIAA1522* gene. We also developed a user-friendly data portal to benefit the biomedical research community.

## Results

### Comprehensive characterization of the transcript landscape across over 1000 cancer cell lines

To extensively dissect the transcriptional atlas across pan-cancer cell lines at the transcript level, reference-based transcript assembly was performed across pan-cancer cell lines (see “Methods”). Briefly, 1017 transcriptomes of cancer cell lines derived from 25 different lineages (Supplementary Fig. 1a and Supplementary Data 1) were subjected to two-round alignments to identify all possible splicing junctions (Supplementary Fig. 1b). Based on this comprehensive repertoire of splicing junctions, expressed transcripts derived from various genomic regions were assembled and quantified. In total, 498,255 transcripts were detected in at least one cell line. On average, 72.31% of transcripts showed expression levels lower than 0.1 TPM, and 11.64% were found to be expressed at higher than 1 TPM (Supplementary Fig. 1c). Among all detected transcripts, 27.24% were detected in less than 10% of all cell lines, while 19.78% were expressed in more than 90% of cell lines (Supplementary Fig. 1d). Except for non-coding and intergenic RNAs, which are likely uncharacterized or small regulatory RNAs, most transcripts from non-coding genomic regions have a length distribution similar to those from protein-coding regions (Supplementary Fig. 1e).

**Fig. 1:**
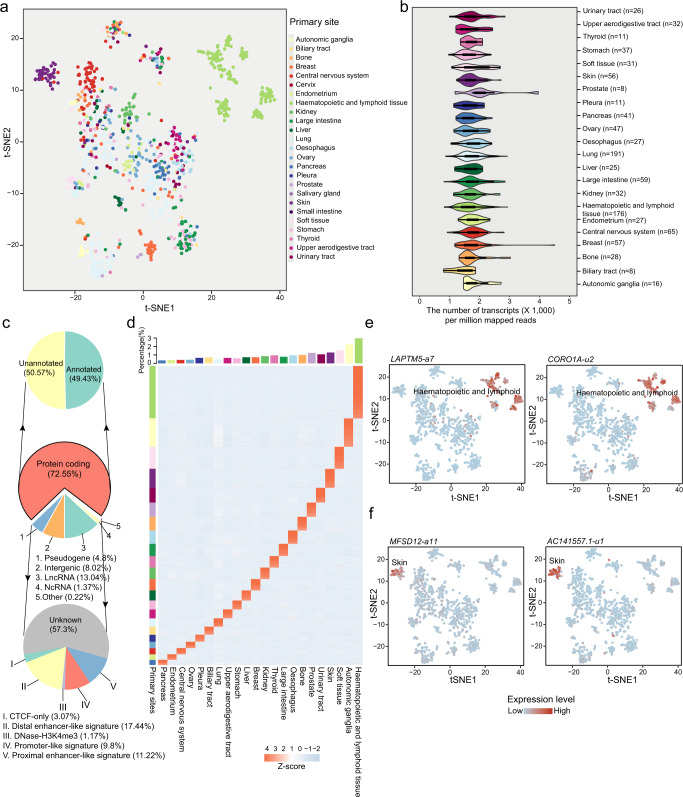
Transcriptional atlas across pan-cancer cell lines. **a** t-SNE clustering based on the expression profile of all detected transcripts across all cell lines. **b** The distribution of normalized transcript numbers across different cell lineages. In each primary site, n is the number of different cell lines. Each box (inset) represents the IQR and median of cell line number in each primary site, whiskers indicate 1.5 times the IQR. **c** Pie charts show the percentages of distinct gene/genomic regions where transcripts are transcribed. The middle pie chart shows different types of genes from which transcripts were derived. The upper pie shows the composition of annotated and unannotated transcripts derived from protein-coding genes. The bottom pie shows the portions of transcripts from different *cis*-regulatory elements (CREs). **d** Lineage-specific transcript profile across cell lineages. Bar plots in the upper panel show the percentage of lineage-specific transcripts in each cell lineage. **e** t-SNE plots show the expression of *LAPTM5-a7* and *CORO1A-u2* transcripts across all cell lines. The specifically highly expressed cell lines are haematopoietic and lymphoid cell lines. **f** t-SNE plots show the expression of *MFSD12-a11* and *AC141557.1-u1* transcripts across all cell lines. The cell lines with specific high expression are skin cell lines. In (**e**) and (**f**), the colors of dots indicate different expression levels of transcripts, wherein a deeper red color represents a higher expression level.

Transcript expression profiles were then adopted to cluster all cell lines, wherein cell lines from the same or close primary sites were clustered closer (Fig. 1a). The numbers of identified transcripts ranged vastly across different cell lines, with the largest median number of 2062 (per million mapped reads) in prostate and the smallest median number of 1472 (per million mapped reads) in biliary tract cell lines (Fig. 1b). Newly assembled transcripts were compared to those annotated in various databases/datasets to filter unannotated transcripts (see “Methods”), removing 35,986 transcripts from the unannotated transcripts (Supplementary Fig. 2a). The vast majority (72.55%) of transcripts were expressed from protein-coding genes (Fig. 1c, Supplementary Data 2). Among all detected transcripts from protein-coding regions, approximately half (50.57%) were unannotated. Moreover, a considerable portion of transcripts are from non-coding genomic regions, such as lncRNAs (13.04%) and pseudogenes (4.81%). The numbers of lineage types that expressed individual transcripts showed a hump distribution, wherein most transcripts were identified in 22 different lineages or one specific cell line lineage (Supplementary Fig. 2b).

The lineage specificity scores were then calculated to identify cancer cell line lineage-specific transcripts (see “Methods”). In total, we identified 72,865 lineage-specific transcripts across 22 different lineages. We further evaluated the specificity of host genes that generated these lineage-specific transcripts. We found that the majority of lineage-specific transcripts were generated from non-specific host genes (Supplementary Fig. 2c). The intergenic and long non-coding RNA transcripts showed the highest overall specificity scores, followed by pseudogene and non-coding RNA transcripts (Supplementary Fig. 2d). The numbers of lineage-specific transcripts ranged from 1201 in the pancreas to 12,769 in haematopoietic and lymphoid cell lineages (Fig. 1d, Supplementary Data 3). For example, the *LAPTM5-a7* transcript (transcribed from the *LAPTM5* gene) and *CORO1A-u2* transcript (an unannotated transcript from the *CORO1A* gene) are exclusively expressed in cancer cell lines derived from haematopoietic and lymphoid lineages (Fig. 1e). One transcript of the *MFSD12* gene, *MFSD12-a11*, and the unannotated *AC141557.1-u1* transcript from the *AC141557.1* gene showed specific transcriptional activities in skin cell lines (Fig. 1f).

We further analyzed available long-read RNA-seq datasets (see “Methods”). In total, 6.23% of our unannotated transcripts were mapped by long-read RNA sequencing reads (Supplementary Fig. 3a). Unannotated transcripts with high expression level were more likely to be mapped by long-read RNA-seq data, wherein 18.23% of the transcripts that showed top 10% expression levels were found to be overlapped by long-read RNA-seq reads (Supplementary Fig. 3b). The coverage of transcriptome by long-read RNA-seq might be lower than that of short-read RNA-seq. In particular, less than 8% of annotated transcripts were covered by long-read RNA-seq reads, while appropriately 40% of them were mapped by short-read RNA-seq reads (Supplementary Fig. 3c). Therefore, the validation percentages of unannotated transcripts by long-read RNA-seq reads in our study were acceptable and reasonable. In a previous study that used short-read RNA-seq data for transcriptome assembly, 7.6% of their newly assembled single-exon transcripts were mapped by long-read RNA-seq reads^24^. To provide more transcription evidence of our unannotated transcripts, we analyzed the CAGE sequencing data from the FANTOM project^25^, and the chromatin states from the Roadmap Epigenomics project^26^. In total, 78.64% of unannotated transcripts were overlapped by transcription evidence (8.28% by only CAGE, 24.08% by only active chromatin states, and 46.29% by both CAGE and active chromatin states), which was comparable with the annotated transcripts, 86.62% of which were mapped by transcription evidence (Supplementary Fig. 3d). In summary, our results presented an extended compendium of the cancer transcriptome and revealed many unexplored RNA transcripts.

### Transcript-level analysis reveals largely unexplored trove in cancer transcriptome

**Fig. 2:**
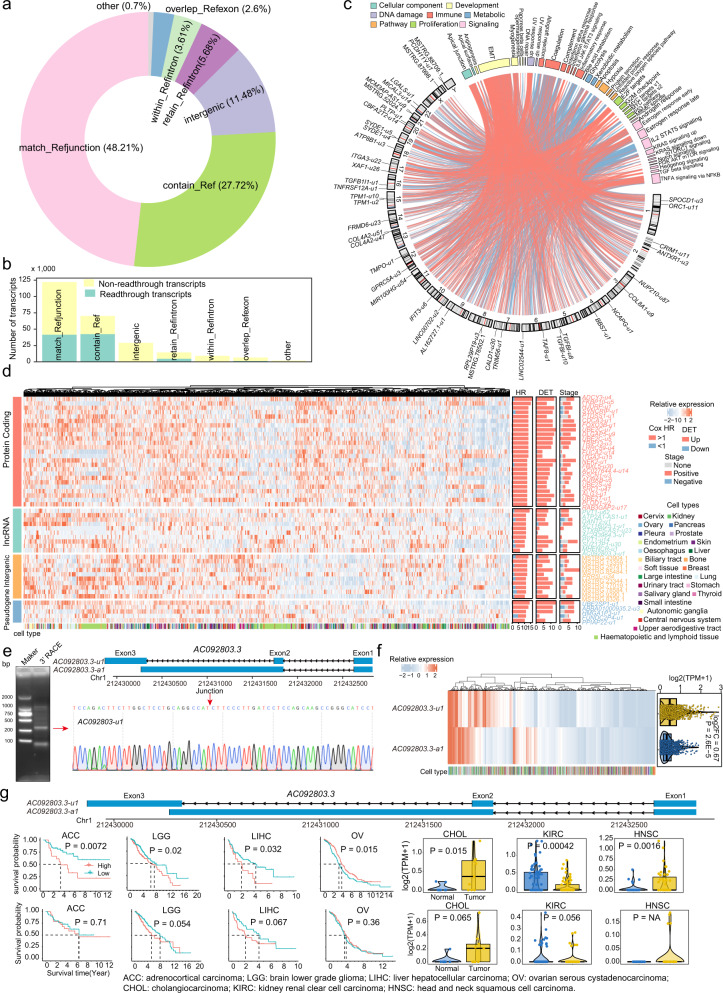
Characterization of unannotated transcripts identified in cancer cell lines. **a** Composition of different unannotated transcript types. match_Refjunction: multi-exon with at least one junction match; contain_Ref: containment of reference (reverse containment); intergenic: no overlap with annotated genes; retain_Refintron: retained intron(s), all or partial introns matched or retained; within_Refintron: fully contained within a reference intron; overlap_Refexon: other same strand overlap with reference exons. **b** Bar plots show the numbers of transcripts matching one single genes (non-readthrough transcripts) or matched more than one gene (readthrough transcripts). **c** Associations between unannotated transcripts and hallmarks. Color bars represent different hallmarks, and bar lengths indicate the number of associated unannotated transcripts. The characters in inner circle indicate the chromosomes. Red links indicate positive correlations, while blue links indicate negative correlations. **d** The heatmap shows representative unannotated transcripts from different gene types, including protein-coding genes, lncRNAs, intergenic genes, and pseudogenes. Bar plots on the right represent the numbers of significant cancer types for each transcript in the Cox proportional hazards model, differential expression analysis, and association analysis of tumor stages. **e** Identification of the unannotated transcript *AC092803.3-u1* in A2780 cells by 3′ RACE and Sanger sequencing. **f** Comparison of the expression levels of *AC092803.3-u1* and *AC092803.3-a1* across cancer cell lines (*n* = 1017). *P*, two-sided Wilcoxon’s rank-sum test *p*-value. Each box represents the IQR and median of expression for each transcript, whiskers indicate 1.5 times IQR. **g** Comparison of survival risk and expression levels between *AC092803.3-u1* and *AC092803.3-a1* transcripts in individual tumor types (*n* = 9 paired tumor and normal samples for CHOL, *n* = 72 for KIRC, *n* = 43 for HNSC). Each box represents the IQR and median of expression in each sample group, whiskers indicate 1.5 times IQR. Log-rank test for survival analysis, two-sided Student’s t test for differential expression analysis.

As unannotated transcripts constituted over half of our cancer transcript atlas, we next investigated whether the expression of these unannotated transcripts was associated with disease progression or prognosis in human cancer. In total, 253,254 unannotated transcripts were identified. Unannotated transcripts from protein-coding genes exhibited relatively lower expression level, while those from the other gene types showed comparable expression levels over annotated transcripts across different expression ranges (Supplementary Fig. 4a). The majority of unannotated transcripts were derived from alternative splicing junctions of multiple exons with at least one annotated junction (Fig. 2a and Supplementary Fig. 4b). In addition, approximately one-third (35.46%) of unannotated transcripts were readthrough transcripts (Fig. 2b). To investigate the possible biological functions that unannotated transcripts may participate in, we performed correlation analysis between unannotated transcripts and transcriptional activities of hallmark biological processes. Significant hallmarks (FDR < 0.05) with the highest correlation were linked to the corresponding transcripts. Epithelial–mesenchymal transition (EMT) was found to be associated with the largest number of unannotated transcripts (Fig. 2c and Supplementary Fig. 5a). The expression levels of individual unannotated transcripts varied widely across different cell lines (Fig. 2d). Compared to paired adjacent non-tumor samples, 75,343 unannotated transcripts showed significantly differential expression in at least one tumor type, wherein LUSC had the largest number of differentially expressed transcripts (Supplementary Fig. 5b). Most of these differentially expressed transcripts showed specific differences in one or two cancer types (Supplementary Fig. 5b). For example, the *UBE2C-u5* transcript showed significant upregulation in 11 different cancer types (Supplementary Fig. 5c). Furthermore, 119,212 showed significant association with tumor stages (Supplementary Fig. 6a), and 131,506 unannotated transcripts were found to be significantly associated with the overall survival of tumor patients in at least one tumor type (Supplementary Fig. 6b). Higher expression of *UBE2C-u5* also indicated more advanced tumor stages (Supplementary Fig. 7a) and poorer prognosis (Supplementary Fig. 7b) across different cancer types.

Unannotated transcripts from protein-coding gene regions exhibited relatively lower expression level, while those from the other gene types showed comparable expression level over corresponding annotated transcripts across different expression ranges (Supplementary Fig. 3a). Comparable expression levels indicated that these unannotated transcripts from non-protein-coding regions might possess alternative or stronger functions over known transcripts of the same host genes in cancer. LncRNAs have been demonstrated to have key roles in human cancer^27,28^, which are also the vast majority of non-protein-coding regions where our unannotated transcripts are from. To select stable and representative unannotated transcripts from lncRNAs for validation, we first ranked the expression levels of those that have no overlaps with protein-coding genes and ≤1000 bp in length (Supplementary Fig. 8a). We also calculated the number of cancer types that unannotated transcripts had survival significance but the corresponding annotated transcripts didn’t. RACE assays were performed to validate the top 10 unannotated transcripts with high average expression level. The unique sequences or junctions of three unannotated transcripts, including *CRIM1-DT-u1*, *AC107032.2-u1*, and *AC092803.3-u1*, were confirmed by 3′ RACE and Sanger sequencing (Supplementary Fig. 8b). Of the three transcripts, *AC092803.3-u1* had the largest number of cancer types where only unannotated transcripts had survival significance. The *AC092803.3-u1* transcript was overlapped by 2, 13, and 4 long-read RNA-seq reads in the K562, PC9, and CACO2 cell lines, respectively (Supplementary Fig. 8c) We also validated the expression of *AC092803.3-u1* in cancer tissue samples, wherein *AC092803.3-u1* showed significantly higher expression than the corresponding annotated transcript *AC092803.3-a1* (*P* = 0.022) (Supplementary Fig. 8d).

The *AC092803.3-u1* transcript was found to be transcribed from the *AC092803.3* gene, which was derived from splicing and joining of 3 exons, two of which were uncharacterized and quite different from the annotated transcript *AC092803.3-a1* (Fig. 2e). The junction that joins exon 2 and exon 3 of *AC092803.3-u1* was not found in *AC092803.3-a1*. The junction of uncharacterized exons 2 and 3 was further validated by 3′ RACE and Sanger sequencing, which demonstrated the valid expression of the *AC092803.3-u1* transcript (Fig. 2e). The *AC092803.3-u1* transcript showed a significantly higher expression level than the other transcript (*P* = 2.6E−5), *AC092803.3-a1*, across 1017 cell lines (Fig. 2f). The expression level of *AC092803.3-u1* significantly distinguished tumor patients with longer survival times from those with shorter survival times, while *AC092803.3-a1* exhibited no association with patient survival in adrenocortical carcinoma (ACC), brain lower grade glioma (LGG), liver hepatocellular carcinoma (LIHC), and ovarian serous cystadenocarcinoma (OV) cohorts (Fig. 2g). Compared to that in paired non-tumor samples, *AC092803.3-a1* showed no expression difference in tumor samples, whereas *AC092803.3-u1* was found to be significantly differentially expressed in cholangiocarcinoma (CHOL, *P* = 0.015), kidney renal clear cell carcinoma (KIRC, *P* = 0.00042), and head and neck squamous cell carcinoma (HNSC, *P* = 0.0016) cohorts. These results indicate that a large number of transcripts with considerable expression levels and valuable clinical significance remain unexplored in the cancer transcriptome.

### Establishment of a high-confidence RBP-transcript regulatory network

**Fig. 3:**
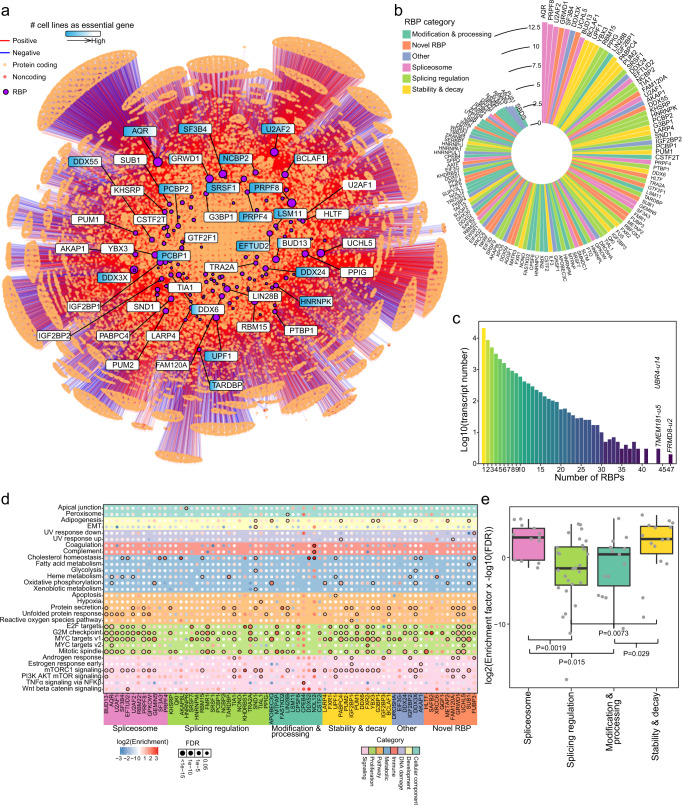
The regulation of transcripts by RBPs. **a** The regulatory network of RBPs and transcripts. **b** The numbers of regulated transcripts for each RBP. **c** The numbers of transcripts regulated by different numbers of RBPs. **d** The bubble plot shows the enrichment of regulated transcripts in biological hallmarks across different RBPs. Bubbles with FDR values <0.05 are labeled with black borders. **e** Comparisons of enrichment among different categories of RBPs (*n* = 13 RBPs for “spliceosome” category, *n* = 28 for “splicing regulation” category, *n* = 15 “modification & processing” category, *n* = 15 for “stability & decay” category). Each box represents the IQR and median of enrichment scores for each category, whiskers indicate 1.5 times IQR. *P*, two-sided Wilcoxon’s rank-sum test *p*-value.

RBPs have been shown to extensively participate in the regulation of post-transcriptional modifications, thus modulating the expression levels of transcripts^29,30^. We next aimed to establish RBP regulation relationships at the transcript level. The RBP knockdown (KD-RNA-seq) and binding (eCLIP-seq) data were integrated to identify high-confidence RBP-transcript regulatory pairs (see “Methods”). A high-confidence regulatory network that consisted of 129 different RBPs and 47,667 different transcripts was constructed (Fig. 3a, Supplementary Data 4), wherein the numbers of up- and down-regulated transcripts had no obvious difference for each RBP (Supplementary Fig. 9a, Supplementary Data 4). We further analyzed the essentiality of RBPs in cancer cells (see “Methods”). Over half of these RBPs (70, 53.85%) showed essentiality in no more than 68 of all examined cancer cell lines (Supplementary Fig. 9b). RBPs that are essential genes in most cell lines also showed a broad range of dependency scores across different cancer cell lines (Fig. 3a and Supplementary Fig. 9c). These RBPs might be important targets for cell viability. Our analysis suggested that RBPs could be targeted to modulate cell viability in specific cell types.

The numbers of regulated transcripts varied largely among different RBPs, with the largest number for AQR (8,955 transcripts) and the smallest number for SBDS (2 transcripts) (Fig. 3b). RBPs were categorized according to their primary functions^31,32^, including “spliceosome”, “splicing regulation”, “modification & processing”, “stability & decay”, “other” and “novel RBP”. RBPs in the “spliceosome” and “stability & decay” categories regulate many more transcripts than those in other categories (Supplementary Fig. 9d). The major portion of transcripts were regulated by a very small number of different RBPs; for example, 21,101 (44.27%) transcripts were regulated by only one RBP, and 8754 (18.36%) transcripts were regulated by two different RBPs (Fig. 3c). However, some transcripts were regulated by many different RBPs, such as the *FRMD8-u2* transcript, which was regulated by 47 different RBPs. Most of the RBPs regulated transcripts that were involved in proliferation, especially “G2M checkpoint”, “MYC targets”, and “E2F targets” (Fig. 3d). Some RBPs regulated transcripts with specific biological functions; for example, the regulation of NOL12 was significantly enriched in “Coagulation” and “Complement”. Compared to those regulated by RBPs that were related to “splicing regulation” and “modification & processing”, transcripts regulated by “spliceosome”- and “stability & decay”-associated RBPs were significantly enriched in “MYC targets v1” (Fig. 3e). In summary, our analysis revealed a fine RBP-transcript regulatory network that might guide transcript manipulation through specific RBPs.

Some human RBPs have been shown to express in a tissue- or cancer-type specific manner, such as ELAVL3 and ELAVL4 in the neuron^33^. Additionally, RBPs that support basic cellular functions are widely expressed across tissues, such as ribosomal and spliceosome RBPs^29^. To extensively examine the expression specificity of RBPs, we collected 1751 RBPs from previous studies^29,31,34^, and calculated a specificity score for each RBP in the RNA-seq datasets of the CCLE, TCGA and GTEx project. We employed the Shannon entropy method to calculate specificity score as described in previous studies^29,35^. In total, 97 (5.57%), 106 (6.09%), and 128 (7.38%) cancer/tissue-type specific RBP genes (specificity score >1) were identified in the CCLE (Supplementary Fig. 10a and Supplementary Data 5), TCGA (Supplementary Fig. 10b), and GTEx (Supplementary Fig. 10c) datasets, respectively. Our results were consistent with previous investigations of human RBP cell/tissue specificity^29,36^. Of these RBPs involved in our study, *LIN28B* showed specifically high expression in the liver and autonomic ganglia cancer cell lines, *LIN28B* and *IGF2BP1* exhibited exclusively high expression in the testicular germ cell cancer, *LIN28B* and *IGF2BP1* showed exclusively high expression in the testis tissue, and *IGF2BP3* exhibited specifically high expression in the skin and testis tissue. Some RBPs may express specific transcripts in certain cancer/tissue types, which generate tissue/cancer-specific RBP protein isoforms. We next calculated specificity scores of 16,312 transcripts generated from RBP genes. On the whole, 688 (4.82%), 2549 (16.85%), and 1548 (10.36%) cancer/tissue-specific RBP transcripts were identified in the CCLE (Supplementary Fig. 10d), TCGA (Supplementary Fig. 10e), and GTEx (Supplementary Fig. 10f) datasets, respectively. Our analysis revealed that many RBPs might exert tissue/cancer-specific functions by expressing specific transcripts and protein isoforms.

### Exploration of therapeutic relevance of transcripts in cancer

**Fig. 4:**
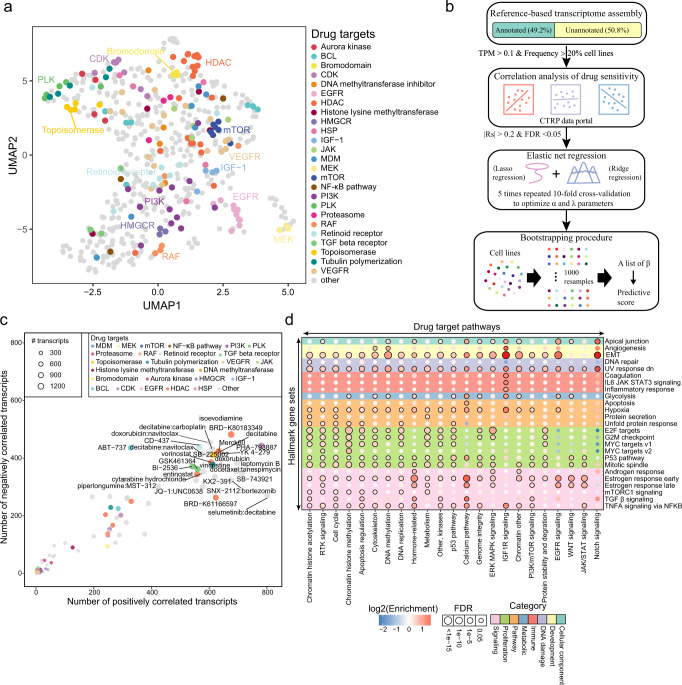
Associations between transcript expression and anti-cancer drug sensitivity. **a** UMAP shows the drug groups clustered by associated transcripts. **b** Workflow of identifying drug-associated transcripts based on a machine learning model. **c** The scatterplot shows the numbers of positively and negatively associated transcripts for each anti-cancer drug (predictive score ≥ 0.7). **d** The bubble plot presents the enrichment of transcripts in hallmarks across different categories of anti-cancer drugs. The X axis represents drug target pathways, and the Y axis represents hallmark gene sets. Bubbles with FDR <0.05 are labeled with black borders.

To further explore the potential clinical utility of transcripts in cancer, associations between transcript expression and anti-cancer drug sensitivity were evaluated. Based on correlations with transcript expression, anti-cancer drugs were clustered, wherein drugs with targets from similar gene classes were more closely clustered, such as HDAC- and EGFR-targeted drugs (Fig. 4a). To further identify the transcripts that were closely associated with the sensitivity to anti-cancer agents, transcripts were first filtered to keep those with considerable abundance across cancer cell lines (Fig. 4b). Then a preliminary correlation between each transcript and drug was evaluated to select possible transcript-drug pairs (see “Methods”). Significant transcript-drug pairs were subjected to an elastic net regression model with 5 rounds of repeated 10-fold cross-validation to optimize the α and λ parameters. The optimized model was submitted to a bootstrapping procedure to generate a predictive score for each transcript-drug pair. Transcript-drug pairs with passing predictive scores (≥0.7) were retained as high-confidence transcript-drug associations. The transcript-drug association network comprised 43,602 (top 1.92%) transcript-drug pairs (Supplementary Fig. 11a). Most of the anti-cancer agents had larger numbers of positively associated transcripts, for example, the sensitivity of PHA-793,887 was positively associated with 781 transcripts and negatively associated with 442 transcripts (Fig. 4c). Moreover, some drugs had balanced positively and negatively associated transcripts, such as ABT-737, which was positively associated with 328 transcripts and negatively associated with 436 transcripts. Most of the anti-cancer agents-associated transcripts were dispersed across various biological processes, while some were notably enriched in specific processes; for example, transcripts that were associated with “Notch signalling”-targeted drugs were specifically enriched in “EMT”, “Apical junction”, and “UV response down” (Fig. 4d). Only “IGF1R signalling”-targeted drugs were associated with transcripts that were involved in immune-related processes, such as “inflammatory response” and “IL6 JAK STAT3 signalling”. Our analysis revealed a large number of anti-cancer drug-associated transcripts that may be used to modulate the response sensitivity of cancer cells to anti-cancer drugs.

### Integrative analysis reveals RBP-transcript-drug axes in cancer

**Fig. 5:**
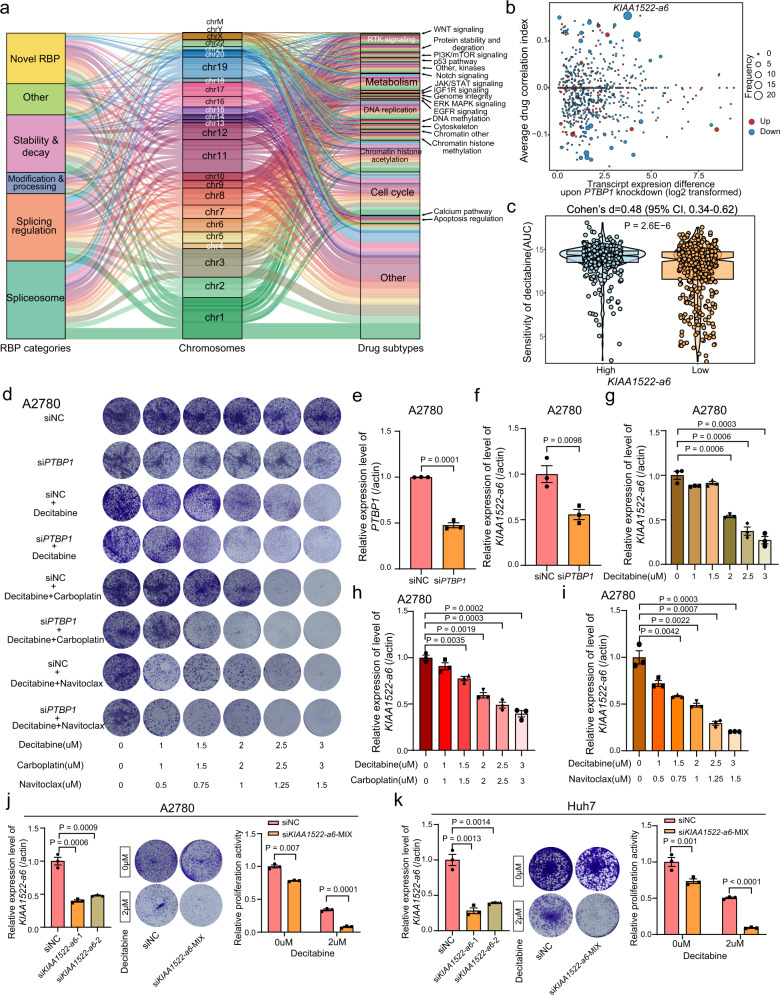
RBP-transcript-drug regulatory axes in cancer. **a** Sankey diagram showing the RBP-transcript-drug regulatory axes in cancer. **b** Drug correlations of differentially expressed transcripts upon the knockdown of *PTBP1*. **c** Comparison of decitabine sensitivity between *KIAA1522-a6* high and low expression cancer cell lines (*n* = 392 for high expression cell lines, *n* = 393 for low expression cell lines). Each box represents the IQR and median of AUC values for each cell group, whiskers indicate 1.5 times IQR. *P*, two-sided Wilcoxon’s rank-sum test. **d** Crystal violet staining of the colony formation assay indicates the sensitivity of siNC-transfected or si*PTBP1*-transfected cells to decitabine, decitabine combined with carboplatin, or decitabine combined with navitoclax. The effect of treatments is shown for A2780 cells. qRT-PCR assays were applied to analyse the expression level of *PTBP1* (**e**) and *KIAA1522-a6* (**f**) after transfection by si*PTBP1* for 48 h in A2780 cells. qRT-PCR assays were applied to analyze the expression level of *KIAA1522-a6* in A2780 cells treated with Decitabine (**g**), Decitabine combined with Carboplatin (**h**), and Decitabine combined with Navitoclax (**i**). The relative expression of *KIAA1522-a6* upon si*KIAA1522-a6*-1 and si*KIAA1522-a6*-2 transfection and the relative proliferation activity upon si*KIAA1522-a6*-MIX and decitabine treatment in the A2780 cell line (**j**) and the Huh7 cell line (**k**). Si*KIAA1522-a6*-MIX is the mixture of si*KIAA1522-a6*-1 and si*KIAA1522-a6*-2. *P*, two-sided Student’s t test *p*-value. *n* = 3 biologically independent samples. Error bars represent the means ± SDs.

The RBP-transcript regulation and transcript-drug association inspired us to propose that RBPs might affect anti-cancer drug sensitivity by regulating drug-associated transcripts. Next, we performed an analysis to integrate the RBP-transcript and transcript-drug networks. Integrative analysis revealed 1,066,380 RBP-transcript-drug axes, bridging 128 RBPs and 430 anti-cancer drugs through 15,511 transcripts (Fig. 5a). The “spliceosome”-, “splicing regulation”-, and “stability & decay”-related RBPs tended to affect sensitivity to more anti-cancer drugs (Supplementary Fig. 11b, c). We next examined the transcripts that were regulated by one specific RBP. Among these transcripts, *KIAA1522-a6* had the second largest number of associated anti-cancer drugs, and showed large expression change upon PTBP1 knockdown (Supplementary Fig. 12a). PTBP1 has been demonstrated to be extensively involved in the regulation of alternative splicing^37^. Knockdown of PTBP1 significantly induced upregulation of 175 and downregulation of 552 transcripts in cancer cells (Fig. 5b). Among them, the *KIAA1522-a6* transcript from the cancer-related protein-coding gene *KIAA1522*^38–41^ had the most connections with anti-cancer drugs, whose higher expression was significantly associated (*P* = 2.6E−6) with a lower response sensitivity to decitabine (Fig. 5c). In the KD-RNA-seq data, a notable decrease of *KIAA1522-a6* was observed upon *PTBP1* knockdown (Supplementary Fig. 12b). The eCLIP binding signals were also observed in multiple exons of the *KIAA1522-a6* transcript. Two specific siRNAs targeting *PTBP1* were designed, si*PTBP1*-1 and si*PTBP1*-2, and both showed efficient knockdown of *PTBP1* in the A2780 and Huh7 cell lines (Supplementary Fig. 13). Compared to that with only *PTBP1* knockdown or decitabine treatment, cell viability notably decreased upon the combination of *PTBP1* knockdown and decitabine treatment (Fig. 5d, e). Furthermore, cancer cells died much more quickly with the knockdown of *PTBP1* and combinational treatments of decitabine and carboplatin or navitoclax. These results indicated that *PTBP1* knockdown could promote the sensitivity of cancer cells to decitabine and combination treatment with decitabine and carboplatin or navitoclax. To further validate that *PTBP1* might impact decitabine sensitivity through the *KIAA1522-a6* transcript, we first examined the expression levels of *KIAA1522-a6*, which showed significant downregulation upon *PTBP1* knockdown (Fig. 5f). The transcriptional activity of *KIAA1522-a6* markedly decreased upon decitabine treatment (Fig. 5g) and combination treatment with decitabine and carboplatin (Fig. 5h) or navitoclax (Fig. 5i). The *PTBP1*-*KIAA1522-a6*-decitabine axes was also demonstrated with the si*PTBP1*-2 (Supplementary Fig. 14a–g), and in the Huh7 cell line (Supplementary Fig. 14h–n). To further explore the causality of the *KIAA1522-a6*-decitabine axis, we knocked down *KIAA1522-a6* in cells treated with 0 µM and 2 µM decitabine. Our results showed that *KIAA1522-a6* knockdown significantly increased the sensitivity to decitabine in the A2780 (Fig. 5j) and Huh7 (Fig. 5k) cell lines. Our integrative analysis uncovered RBP-transcript-drug axes that might provide potential treatment strategies in cancer.

### TAiC: a user-friendly data portal for the transcript atlas in cancer

**Fig. 6:**
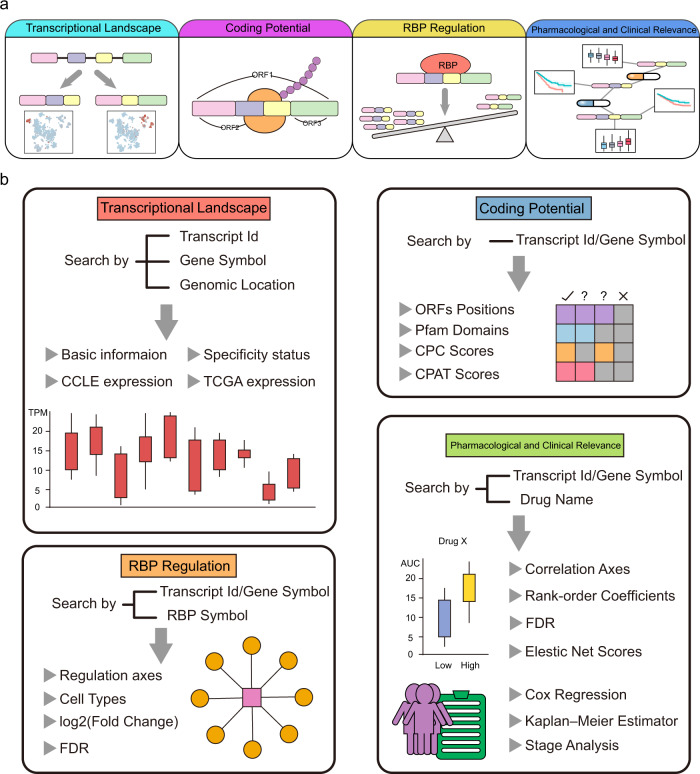
Diagram of the TAiC data portal. **a** Overall design of the TAiC data portal. **b** The full resource of TAiC is available to search, browse, visualize, and download. TAiC offers access to the transcriptional atlas of transcripts, coding potential of unannotated and non-coding transcripts, RBP-transcript regulation, transcript-drug and clinical associations.

To promote the exploration of molecular troves across over 1000 human cancer cell lines at transcript-level resolution, we developed a comprehensive and interactive web resource, the Transcript Atlas in Cancer (TAiC, http://www.shenglilabs.com/TAiC/). In this data portal, we provide four interactive modules, including the expression landscape of transcripts, coding potential of unannotated and non-coding transcripts, RBP-mediated transcript expression, and pharmacological and clinical relevance (Fig. 6a). Users can query and visualize the expression level of individual transcripts in multiple cancer cell lines (Fig. 6b). TAiC enables users to examine the RNA sequences and coding potential of unannotated RNAs. TAiC also provides an RBP-transcript regulatory network for users to explore the upstream regulating factors of transcript expression across cancer cell lines. Users can also investigate the associations between transcript expression and anti-cancer drug sensitivity. In addition, users can also explore the expression changes and clinical relevance of transcripts in 33 different cancer types. TAiC will be continuously updated to serve as an instructive resource for researchers to investigate cancer at the transcript level.

## Discussion

With the rapid development of high-throughput RNA sequencing techniques and computational algorithms, transcriptome diversity has been gradually realized in complex human diseases, including cancers. By employing reference-based transcript assembly in more than 1000 cancer cell lines, we presented a comprehensive human cancer transcriptome. Due to the limitation of available large-scale RNA-seq data, the majority of non-polyadenylated RNA transcripts were not detected in our study. However, our results covered major RNA biotypes, which also largely complemented other efforts aiming to annotate human transcripts.

Lorenzi et al. presented a comprehensive atlas of the human transcriptome through transcriptome assembly of 300 human tissues and cell lines^24^. Our study is quite different from this study. Firstly, our study is more focused on cancer transcriptome. This study only included 89 cancer cell lines, while we examined 1017 cancer cell lines and investigated the clinical significance of transcripts in cancer. Secondly, this study focused on non-coding RNAs, whereas we examined all detected transcripts, including those from protein-coding gene, lncRNAs, and other non-coding regions. Thirdly, we also built the RBP-transcript regulatory network and the associations between transcripts and the response of cancer cells to anti-tumor drugs. Other studies of large-scale transcriptome assembly used other RNA-seq datasets and focused on different aspects of transcriptome. Jiang et al.^42^ and Iyer et al.^43^ presented expanded landscapes of lncRNAs from thousands of RNA-seq data of tumor, normal, and cell line samples. Based on transcriptome assembly from RNA-seq datasets of 768 patient samples in TCGA datasets, Attig et al. uncovered a large number of long-terminal repeat (LTR)-overlapping transcripts^44^. Pertea et al. constructed an expanded human gene catalog from deep RNA-seq libraries of nearly 10 thousand normal samples^45^.

In the present study, approximately 50% of the detected transcripts were unannotated. This may be due to the reference-based transcriptome assembly strategy used in this study. In addition to existing junctions, our analysis identified many unannotated junctions in both known genes and uncharacterized genomic regions. Of note, approximately one-third of unannotated transcripts spanned more than one gene, named readthrough transcripts. Readthrough transcripts are RNA molecules that are generated through the splicing of exons from multiple distinct genes^46^, which is quite common in human transcriptome^27,47^. Most previous studies have focused on gene levels that were combinations of all transcripts from the same genes, which only quantified reads falling within genomic regions of reference genes^48^. Our previous studies based on RNA-seq data also identified many unannotated transcripts that were further validated to play important roles in cancer^10,34^. Lorenzi et al. reported thousands of uncharacterized non-coding RNAs from RNA-seq data^24^. These studies demonstrated that a considerable part of the human transcriptome remains uncharacterized. The uncharacterized transcriptome has been concealed, at least in part, by reference-based transcriptome quantification. Together with these reports, our study provides an important complement to the existing human transcriptome. In the RBP-transcript network, 62.43% of RBP-regulated transcripts were unannotated (Supplementary Fig. 15a). The unannotated transcripts occupied over half of regulated transcripts by each RBP (Supplementary Fig. 15b). In the RBP-transcript-drug axes, 53.78% of all transcripts that bridged RBPs and drugs were unannotated (Supplementary Fig. 15c). Furthermore, the unannotated transcripts linked 75.27% RBP-drug connections together with annotated transcripts, and 15.97% of the RBP-drug connections was linked by only unannotated transcripts (Supplementary Fig. 15d). These results showed that unannotated transcripts contributed appropriately half of the links to the RBP and drug networks.

Various strategies of selecting unannotated transcripts for validation can be applied to choose transcripts of interest, such as expression levels, specific cell types, clinical significance, specific gene types, or coding potential. We constructed the TAiC data portal to serve the research community to explore potential functions of these unannotated transcripts. A part of the unannotated transcripts might be incomplete or even nonexistent due to the limitations of short-read RNA sequencing and the transcript assembly algorithm, and cannot be experimentally validated. However, to our best knowledge, our study made nonnegligible contribution to the transcript-level exploration of cancer transcriptome.

The integration of RBP-transcript regulation and transcript-drug association networks enables the identification of RBPs that could affect the sensitivity to anti-cancer drugs by regulating transcript expression. Our analysis linked RBPs to anti-cancer drugs through transcripts. Several RBPs have been demonstrated to mediate drug sensitivity in cancer, such as ERα in breast cancer^14^, CELF2 in ovarian cancer^49^, and hnRNPA0 in p53-mutant tumors^50^. These studies showed that RBPs could be used to modulate the response sensitivity to anti-cancer drugs of cancer cells. Our study provided a resource documenting thousands of RBP-transcript-drug axes, which is expected to offer alternative strategies to modulate drug resistance in cancer.

Third-generation RNA-seq technologies have shown great power to capture and characterize full-length RNA transcripts, such as Nanopore and PacBio long-read RNA sequencing^51^. These long-read RNA technologies could correct a major bias in next-generation RNA-seq data, wherein fragmented sequencing reads were computationally mapped and assembled to refer to original RNA transcripts^48^. With the rapid development of long-read RNA sequencing technology, we believe that more and more unannotated transcripts identified in our study will be functionally validated.

## Methods

### RNA-seq datasets of different cancer cell lines

The raw RNA-seq data of 1017 cancer cell lines were downloaded from the Sequence Read Archive (SRA, https://www.ncbi.nlm.nih.gov/sra) database with the accession number SRP186687 by utilizing the prefetch tool (version 2.10.8) in the SRA Toolkit (http://ncbi.github.io/sra-tools/). Then, FASTQ files of raw RNA-seq reads were extracted from the SRA by using the fasterq-dump tool (version 2.10.8). Detailed information of these cancer cell lines was supplied in Supplementary Data 1.

### Transcript assembly and quantification from RNA-seq data

Raw RNA-seq reads were aligned to the human reference genome (GRCh38, https://www.gencodegenes.org/human/release_35.html) by using STAR software (version 2.7.6a)^52^. To achieve the most sensitive unannotated junction discovery, STAR was run in the 2-pass mode, which allowed more mapping of splicing reads to unannotated junctions. In particular, STAR was run with usual parameters in the first-pass run, wherein the junctions were collected. All detected junctions were subjected to second-pass mapping. The alignments obtained from STAR 2-pass mapping were provided as input to StringTie (version 2.1.4)^53^ for reference-based transcript assembly. Transcript annotation from GENCODE^54^ version 35 (https://www.gencodegenes.org/human/release_35.html) was adopted as the transcript model reference to guide the assembly process with the “-G” option. Transcript assembly was performed separately for each cell line. Then, all transcript assemblies were merged to generate a nonredundant master set of transcripts for all cell lines by using StringTie “–merge” mode. The GffCompare tool (version 0.12.2)^55^ was employed to compare newly assembled transcripts with those annotated in various databases/datasets, including ENCODE^56^, UCSC known genes^57^, RefSeq genes^58^, AceView^59^, CHESS^45^, RefLnc^42^, and LTRs assembled by Attig et al.^44^. The transcripts that were not matched in these databases/datasets were defined as unannotated transcripts in the following analysis. StringTie quantification was utilized to reveal both transcript- and gene-level expression for individual cell lines. Expression levels were normalized in TPM (transcripts per million mapped reads). Transcripts with expression levels ≥0.1 TPM in at least one cell line were retained for subsequent analysis. We used different criteria of transcript expression levels (0.1, 0.5, 1, 2, and 3) and cell line numbers (1, 2, 5, 10, and 20). As expected, the number of unannotated transcripts decreased more with the increasing criteria of cell line numbers (Supplementary Fig. 16). The threshold of TPM 0.1 was shown to be a robust and sensitive expression detection threshold for lowly-expressed transcripts, which has been used in many previous studies^24,60,61^. Transcripts were named by using their respective gene names followed by an “a” or “u” with numbers for annotated or unannotated transcripts, respectively (Supplementary Data 2). The StringTie names were used for those transcripts that overlapped with no known genes.

### Processing long-read RNA-seq data

The available raw long-read RNA-seq data of cancer cell lines were downloaded from the ENCODE data portal, including the A673, CACO2, CALU3, HCT116, HepG2, K562, MCF7, PANC1, PC3, and PC9 cell lines. The raw long-read RNA-seq data were processed by utilizing the FLAIR pipeline (version 1.5)^62^. In particular, reads were aligned to the human reference genome (GRCh38) by using minimap2 (version 2.17-r941)^63^ in spliced alignment mode with default parameters. High-confidence isoforms were then collapsed by the collapse function implemented in FLAIR. The quantify function was employed to quantify transcripts, wherein only reads with alignment scores >1 were used.

### Analysis of transcription evidence for unannotated transcripts

The Cap Analysis Gene Expression (CAGE) sequencing results were downloaded from the FANTOM project^25^. The chromatin states of human genome were obtained from the Roadmap Epigenomics project^26^. Chromatin states indicative for active transcription, including transcription, active transcription start site, transcribed and regulatory, bivalent_promoter, transcribed and enhancer, and active enhancer, were used for the integrative analysis with CAGE data. The presence of CAGE sequencing or chromatin state peaks within 500 nt around the transcription start site (TSS) was considered as transcription evidence of corresponding transcripts.

### Calculation of coding potential of unannotated transcripts

The coding potential of each unannotated transcripts was evaluated by using the CPC2 (version 1.01)^64^ and CPAT (version 3.0.4)^65^ software. CPC2 predicted the coding potential of RNA sequences by a SVM model trained from four intrinsic features, including Fickett TESTCODE score, open reading frame (ORF) length, ORF integrity, and isoelectric point (pI). CPAT uses four sequence features to distinguish coding and non-coding RNA transcripts, including open reading frame (ORF) size, ORF coverage, Fickett TESTCODE statistic, and hexamer usage bias.

### Calculation of transcriptional activity of hallmark biological processes

To estimate the transcriptional activities of hallmark biological processes, gene lists of 50 hallmarks were retrieved from the MSigDB database^66^. For each hallmark, the Gene Set Variation Analysis (GSVA)^67^ algorithm (version 1.38.2) was employed evaluate the overall transcriptional activity in single sample mode. The GSVA method estimates variation of activities of individual gene sets over a sample population in an unsupervised manner. Each sample was endowed 50 activity scores of different hallmarks.

### RACE assays

We performed the RACE analyses to determine full length of the *CRIM1-DT-u1* (*CRIM1-DT-u1*-3′GSP: GGGGCCAGATTGGAGTTCGA), *AC107032.2-u1* (*AC092803.3-u1*-3′GSP: AGGGAAGAGCACTTTGGTCA), and *AC092803.3-u1* (*AC107032.2-u1*-3′GSP: CCTGGTCTGGTCAGGGCTCAGTTAG) transcript by using a SMARTer™ RACE cDNA Amplification Kit (Clontech, California, USA) according to the manufacturer’s instructions.

### eCLIP-seq and knockdown RNA-seq datasets of RNA-binding proteins

We retrieved paired eCLIP-seq and KD-RNA-seq (knockdown followed by RNA sequencing) datasets from the Encyclopedia of DNA Elements project (ENCODE, https://www.encodeproject.org/)^31^, covering 85 and 107 different RBPs in HepG2 and K562 cell lines, respectively. All raw sequencing reads were first subjected to Trimmomatic (version 0.39)^68^ to remove adapters and low-quality bases. For eCLIP-seq data, we followed the ENCODE processing pipeline^31^ to obtain enriched binding regions for each RBP. Trimmed KD-RNA-seq reads were mapped to the human reference genome (GRCh38) by using STAR (version 2.7.6a) in two-pass mode^52^. The alignments were then subjected to StringTie (version 2.1.4)^53^ with our assembled transcriptome to quantify transcript abundance. Finally, DESeq2 (version 1.30.0)^69^ was applied to compare transcript differences between RBP-knockdown and control cell lines for each RBP.

### Estimating the expression specificity of transcripts across pan-cancer cell lines

To obtain lineage-specific transcripts, a specificity score was calculated for each transcript according to a previous study^29^. In particular, the specificity score was equal to the logarithm of the lineage number minus the Shannon entropy of transcript expression. The calculation was conducted as follows:

1 $${S}_{t}={\log }_{2}(N)-\left(-\mathop{\sum }\limits_{i=1}^{N}({p}_{it}\times {\log }_{2}{p}_{it})\right)$$

where *S*_*t*_ represents the specificity score of transcript *t*, *N* is the total number of cell lineages, and *p*_*it*_ indicates the expression ratio of transcript *t* in lineage *i*. One specificity score and *N* expression ratio were assigned to each transcript. The expression ratio of each transcript across all lineages was calculated as follows:

2 $${p}_{it}=\frac{{x}_{it}}{{\sum }_{i=1}^{N}{x}_{it}}$$

where *p*_*it*_ is the expression ratio of transcript *t* in lineage *i*, *N* indicates the total number of lineages, and *x*_*it*_ represents the expression value of transcript *t* in lineage *i*.

When the largest expression ratio was more than two times the second largest expression ratio and the specificity score was larger than 1, the transcript was defined as a lineage-specific transcript in the lineage with the largest expression ratio. The expression specificity of RBPs across cancer cell lines, cancer tissues, and normal tissues was also calculated as described above.

### Construction of an RBP-transcript regulatory network

The KD-RNA-seq and eCLIP-seq data for individual RBPs were subjected to integrative analysis to identify high-confidence RBP-transcript regulatory relations. For each RBP, transcripts with |fold change| > 1.5 and FDR < 0.05 were considered significantly changed upon RBP knockdown. High-confidence RBP-transcript regulatory relationships were established when RBP binding signals were found in these significantly changed transcripts.

### Analyzing the essentiality of RBPs in cancer cells

The gene dependency scores of 17,386 genes across 1086 cancer cell lines were downloaded from the DepMap data portal (https://depmap.org/portal/), which were determined by using high-throughput CRISPR screening. In this dataset, genes with a dependency score < −1 are considered as essential genes in the corresponding cell lines. We extracted the RBPs and cell lines involved in our study, generating a dependency score matrix of 130 RBPs and 671 cancer cell lines.

### Transcript analyses in TCGA datasets

The aligned RNA-seq reads of 33 TCGA cancer types were downloaded from the Genomic Data Commons data portal (GDC, https://portal.gdc.cancer.gov/) with official authorization, including 10,358 samples across 33 cancer types. All alignments were subjected to StringTie (version 2.1.4) with customized transcript annotation to quantify transcript abundance. Normalized expression matrixes were employed to perform differential expression analysis by adopting paired Student’s t test (as implemented in R software). Only cancer types with no <5 paired tumor and adjacent non-tumor samples were involved in this differential expression analysis. Transcripts that were expressed ≥0.1 TPM in no less than 25% of tumor or adjacent non-tumor samples in each cancer type were kept for downstream analysis.

### Survival analysis

Clinical follow-up information (days to last follow-up and vital status) of tumor patients was retrieved from GDC data portal (https://portal.gdc.cancer.gov/)^70^. For each transcript in each tumor type, tumor patients were divided into high- and low-expression groups by using the median expression level of the transcript. Then the overall survival time was compared by using log-rank test implemented in the survival package (version 3.4-0, https://CRAN.R-project.org/package=survival). The survival curves were generated by using the Kaplan-Meier method in the survminer package (version 0.4.9, https://CRAN.R-project.org/package=survminer).

### Associating transcript expression and drug response by elastic net regression

The strategy that combines elastic net regression and bootstrapping was used to evaluate the associations between transcript expression and drug sensitivity as described in a previous study^71^. To feed elastic net regression with a stable transcript expression profile, transcripts that were expressed (TPM > 0.1) in less than 20% of the cell lines were filtered out. The sensitivity (AUC values) to anti-cancer drugs was retrieved from the CTRP database (https://portals.broadinstitute.org/ctrp.v2.1/)^21^, which included 481 compounds across 887 cancer cell lines. Then correlations of all possible transcript-drug pairs were calculated by Spearman correlation. Transcript-drug pairs with Spearman R > 0.2 and FDR < 0.05 were used to build a prediction matrix, *n* × *t*, wherein *n* is the number of cancer cell lines and *t* is the number of transcripts. The prediction matrix was first normalized for each transcript to have zero mean and unit standard deviation. The normalized prediction matrix was then fitted for the elastic net regression by utilized the glment R package (version 4.1)^72^. To minimize the root mean squared error, the caret package (version 6.0–86) was employed to optimize the α and λ parameters. In particular, 10-fold cross-validation was performed 5 times with 25 possible *α* and *λ* values in random search mode.

### Prioritizing transcript-drug pairs through the bootstrapping procedure

To obtain reliable transcript-drug pairs, the bootstrapping procedure was performed with the optimal *α* and *λ* parameters to produce 1000 resampled datasets by sampling with replacement. For each resampled dataset, a list of regression coefficients (*β*) was generated, which was used to calculate a prediction score as follows:

3 $${{{{{\rm{Score}}}}}}=\left\{\begin{array}{c}\frac{F[\beta \, > \, 0]\,-F[\beta \, < \, 0]}{1000}\,{{{{{\rm{if}}}}}}\,F[\beta \, > \, 0] \, > \, F[\beta \, < \, 0]\\ \frac{F[\beta \, < \, 0]\,-F[\beta \, > \, 0]}{1000}\,{{{{{\rm{if}}}}}}\,F[\beta \, > \, 0] \, < \, F[\beta \, < \, 0]\end{array}\right.$$

where F[*β* > 0] represents the frequency of transcripts with positive coefficients in bootstrap datasets, and F[*β* < 0] represents the frequency of transcripts with negative coefficients in bootstrap datasets. Transcripts with a prediction score ≥0.7 were considered significantly predictive of the sensitivity of specific drugs.

### Cell culture

A2780 and Huh7 cells were maintained in DMEM medium supplemented with 10% fetal bovine serum, 100 mg/ml penicillin, and 100 U/ml streptomycin. A2780 cell line was purchased from the American Type Culture Collection (ATCC, Manassas, VA, USA). Huh7 cell line was purchased from the Shanghai Cell Bank Type Culture Collection (Shanghai, Chinese Academy of Sciences, China).

### Transfection of cell lines

SiRNAs and negative control siRNAs were designed and synthesized by RiboBio (RiboBio Biotechnology, Guangzhou, China). Specific RNAi sequences are as follows (5′-3′): si*PTBP1*-1, CAAAGCCUCUUUAUUCUUU; si*PTBP1*-2, CUUCCAUCAUUCCAGAGAA; si*KIAA1522-a6*-1, ACUCACACCACAAGAGGAAG; si*KIAA1522-a6*-2, GUCCCCGGGUCCGCAGCUUC. Cells were transfected with the siRNAs using Oligofectamine transfection reagent (RNAi MAX, Invitrogen) according to the manufacturer’s instructions. The cells were harvested 48 h after transfection for further analysis.

### Cell viability assay

Cell viability was determined by CCK8 assay. Briefly, A2780 and Huh7 cells (5 × 10^3^ cells/well) were seeded into 96-well plates. After 24 h of culture, the cells were treated with carboplatin, decitabine, and navitoclax (MCE, Shanghai, China) at the indicated concentrations for another 24 h. CCK8 solution (10 μl) was added to each well, and the cells were further incubated at 37 °C for 3 h. The absorbance of each well was measured at 450 nm with a spectrophotometer.

### Colony formation assay

A2780 and Huh7 cells transfected with siRNAs were seeded into a 12-well plate and incubated with complete medium at 37 °C for 24 h. Then, the cells were treated with different concentrations of carboplatin, decitabine, and navitoclax (MCE, Shanghai, China) for another 10 days. The cells were fixed with 4% paraformaldehyde and stained with 2% crystal violet. Images were obtained, and the number of colonies was counted. Different concentrations of carboplatin, decitabine, and navitoclax (MCE, Shanghai, China) were diluted in dimethyl sulfoxide (DMSO) (Sigma-Aldrich) or PBS.

### Quantitative reverse transcription PCR

Total RNA was isolated from cells by using TRIzol Reagent (Thermo Fisher Scientific, Massachusetts, USA). Then, the extracted RNAs were reverse transcribed into cDNA by using a Superscript II reverse transcription kit (Takara Bio, Beijing, China) according to the manufacturer’s protocols. Subsequently, qRT-PCR was conducted with a SYBR-Green master kit (Vazyme, Nanjing, China) on a LightCycler 480 II (Roche Diagnostics) instrument according to the manufacturer’s protocols. The primers used to amplify PTBP1 (*PTBP1*_q_F1: CTCCAAGTTCGGCACAGTGTTG; *PTBP1*_q_R1: CAGGCGTTGTAGATGTTCTGCC), *KIAA1522-a6* (*KIAA1522-a6*_q_F1: ACTCACACCACAAGAGGAAG; *KIAA1522-a6*_q_R1: TTTGTCATTCTCAGCCTTGG), and β-actin (β-actin-F: TTGTTACAGGAAGTCCCTTGCC; β-actin-R: ATGCTATCACCTCCCCTGTGTG) were chemically synthesized by TSINGKE (TSINGKE, Beijing, China). All qRT‑PCRs were performed in triplicate.

### Western blot assay

Proteins were subject to SDS-PAGE and transferred to the nitrocellulose membranes (GE, CT, USA). After being blocked by non-fat milk, the membrane was incubated with PTBP1 polyclonal antibody (Proteintech, cat#12582-1-AP) and GAPDH monoclonal antibody (Proteintech, cat#60004-1-Ig). The band density was analyzed using ImageJ and compared with the internal control.

### Database and web site implementation

The TAiC database was built with the Python FLASK_REST API (https://flask-restful.readthedocs.io/) as a backend web framework. In the TAiC database, MongoDB (https://www.mongodb.com) was adopted for data deposition and management. Angular (https://angular.io/) was utilized to develop web interfaces of TAiC. The frontend framework was constructed by using Bootstrap (https://getbootstrap.com). Data visualization was carried out by Echarts (https://echarts.apache.org/). The TAiC online database was tested and found to be supported in popular web browsers, including Microsoft Edge, Google Chrome, Firefox, and Safari. The TAiC database is publicly accessible at http://www.shenglilabs.com/TAiC/.

### Statistics and reproducibility

Statistical analysis and data visualization in this study were performed by using R software (R Foundation for Statistical Computing, Vienna, Austria; http://www.r-project.org). Unless otherwise specified, all tests were two-tailed, and a *P* or FDR value <0.05 was considered to indicate statistical significance. All experiments were repeated independently three times.

### Reporting summary

Further information on research design is available in the Nature Portfolio Reporting Summary linked to this article.

## Supplementary information

Supplementary figures

Description to Additional Supplementary Information

Dataset 1

Dataset 2

Dataset 3

Dataset 4

Dataset 5

Reporting Summary

## Data Availability

The raw RNA-seq data of CCLE project were downloaded from the SRA database (SRP186687). The eCLIP-seq and KD-RNA-seq were retrieved from the ENCODE database (https://www.encodeproject.org/). The sensitivity to anti-cancer drugs of cancer cell lines was retrieved from the CTRP database (https://portals.broadinstitute.org/ctrp.v2.1/). The human reference genome and transcript annotation were downloaded from the GENCODE database (https://www.gencodegenes.org/human/release_35.html). Software and resources used for analysis and plotting are described in each method section. All results generated in this study can be found in supplementary tables and the TAiC data portal (http://www.shenglilabs.com/TAiC/). Source data are provided with this paper.

## Source data

Source Data

## Code availability

Scripts and codes that were used to generate the data in the TAiC data portal were deposited in https://github.com/lishenglilab/TAiC/tree/main/Code.
