# Supplementary material for: Systematic characterization of cancer transcriptome at transcript resolution
Source: Nat Commun. 2022 Nov 10;13:6803. doi: 10.1038/s41467-022-34568-z (PMC9649690; doi:10.1038/s41467-022-34568-z)
Supplement: Supplementary file 1 — Supplementary figures [file 41467_2022_34568_MOESM1_ESM.pdf]

Supplemental Figures

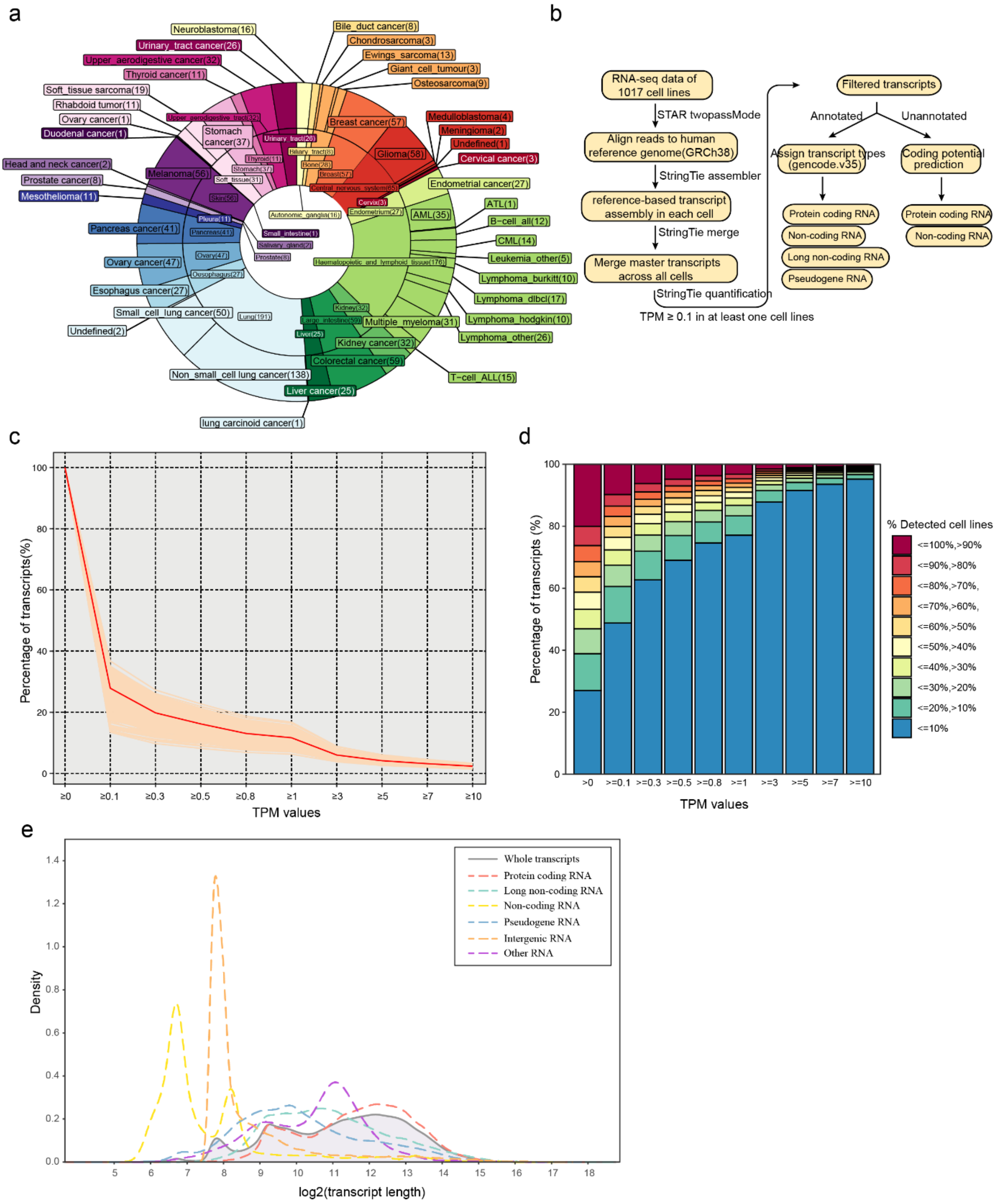

Supplementary Fig. 1 Establish and characterize a comprehensive catalogue of transcripts across pan-cancer cell lines. a The numbers of cell lines in original cancer types and tissues. **b** Flowchart of establishing a comprehensive catalogue of transcripts in ~1,000 cancer cell lines. **c** Overall percentage distribution of transcripts with various expression levels. **d** Cell line frequency distribution of detected transcripts. **e** The length distribution of transcripts across different RNA types.

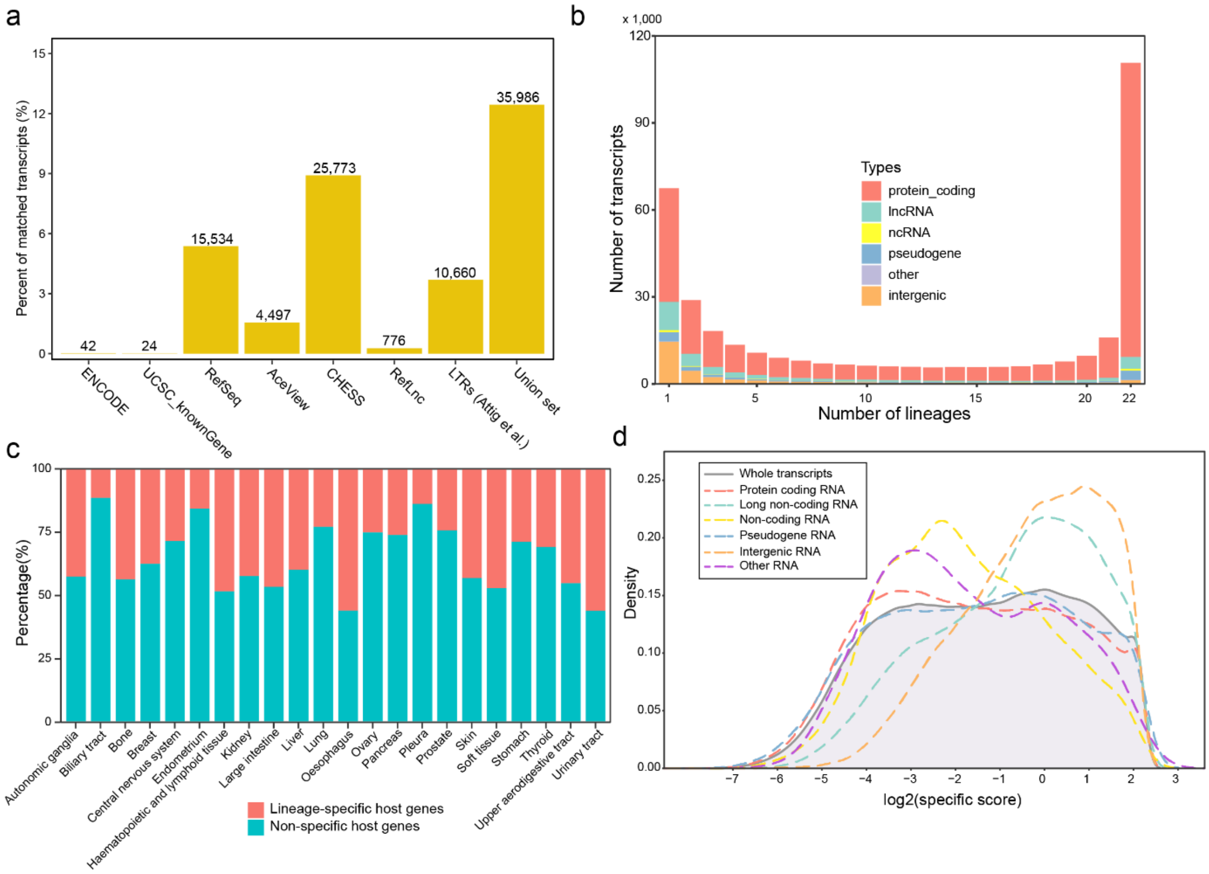

Supplementary Fig. 2 The distribution of transcripts across multiple cell line lineages. a The percentages and numbers of newly assembled transcripts that were matched in different databases/datasets. The last bar represents total matched transcripts. **b** The number distribution of transcripts detected in different amounts of cell line lineages. **c** The percentages of lineage-specific transcripts generated from lineage-specific host genes or non-specific host genes. **d** The distribution of lineage specificity scores in different types of RNA transcripts.

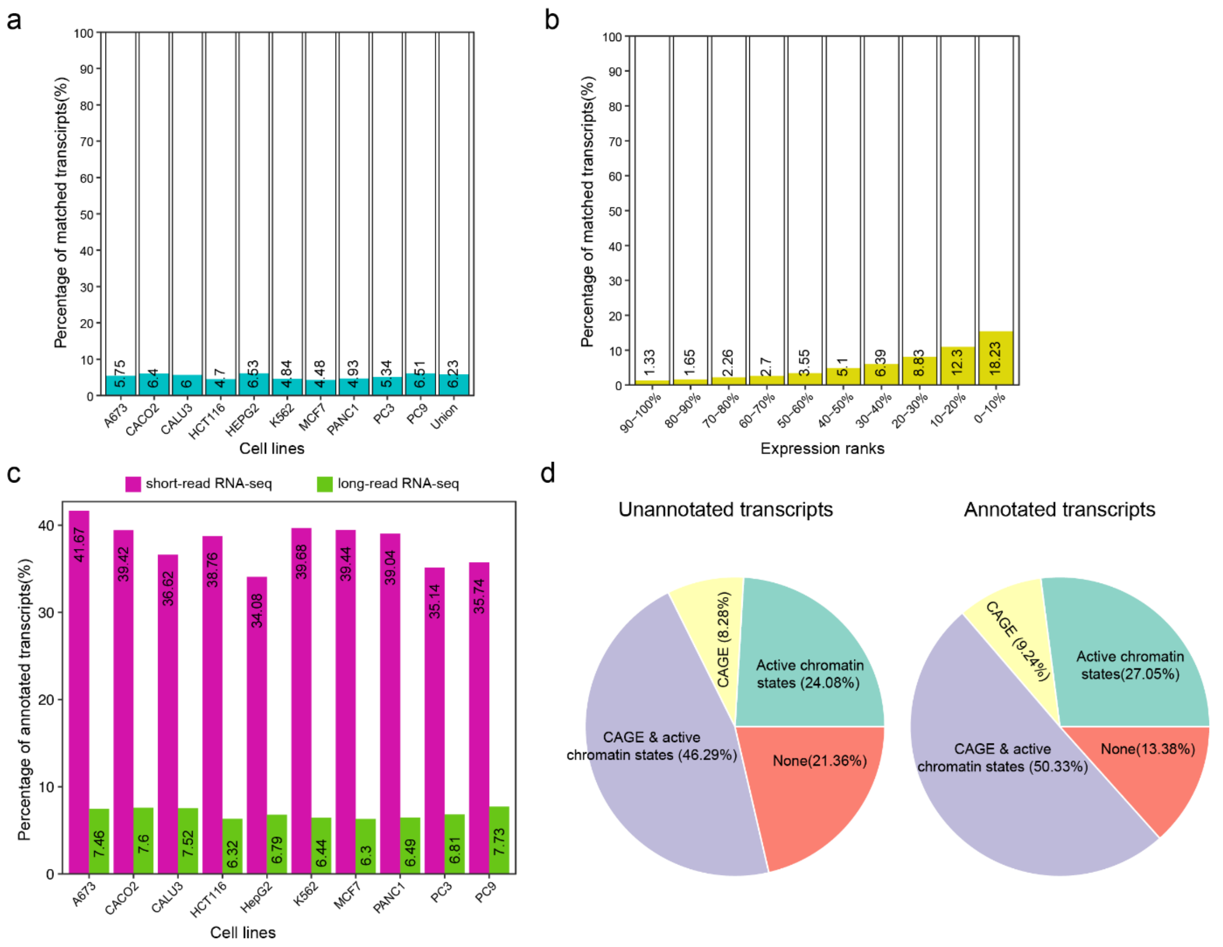

Supplementary Fig. 3 Validation of unannotated transcripts in long-read RNA-seq datasets. a Percentages of matched unannotated transcripts in each long-read RNA-seq dataset. **b** Percentages of matched unannotated transcripts in different ranges of expression levels. **c** Percentages of detected annotated transcripts in each short-read and long-read RNA-seq dataset. **d** Pie charts show the percentages of unannotated (left) and annotated transcripts (right) that have evidence of CAGE only, active chromatin states only, both CAGE and active chromatin states, or none.

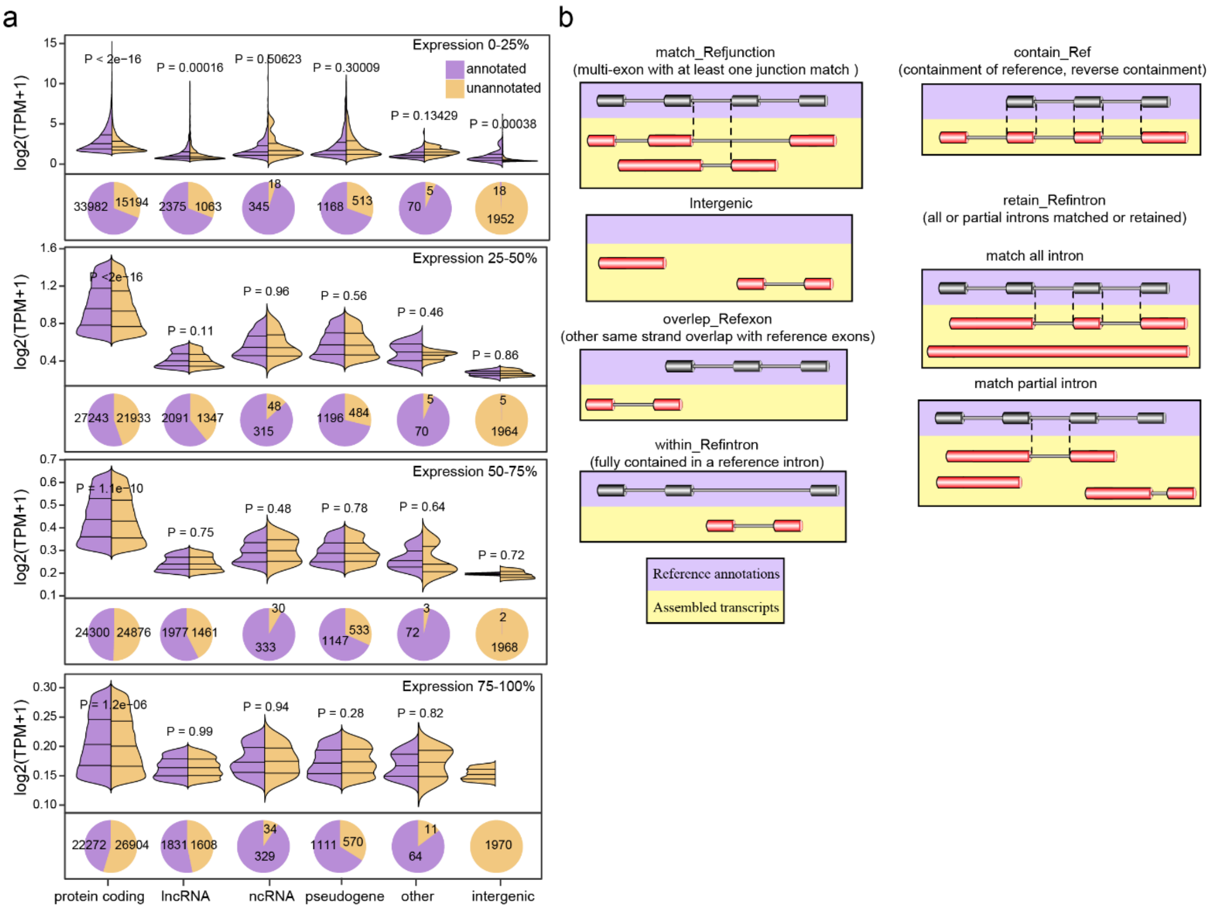

Supplementary Fig. 4 Comparisons between annotated and unannotated transcripts. a Comparisons of expression levels between annotated and unannotated transcripts in different expression ranges. P, two-sided Wilcoxon's rank-sum test p-value. **b** Comparisons of genic models between assembled transcripts and annotated genes.

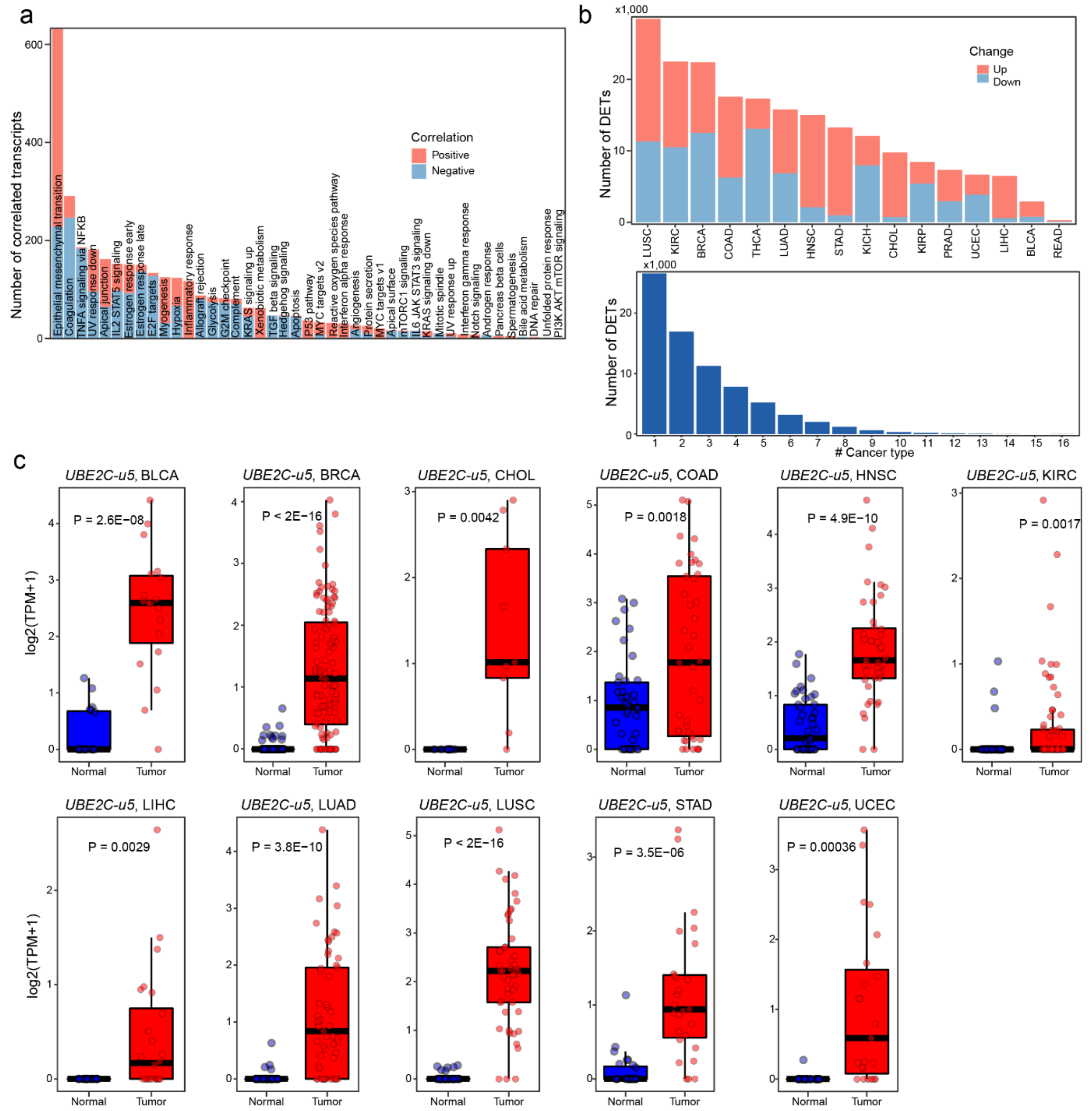

Supplementary Fig. 5 Unannotated transcripts show extensive dysregulation in cancer. a The number of associated unannotated transcripts in each hallmark. **b** The number of differential unannotated transcripts across different cancer types and the number of unannotated transcripts that show differential expression in one or multiple cancer types. **c** Boxplots show comparisons of *UBE2C-u5* transcript between tumor and paired non-tumor samples across different tumor types (n = 19 paired tumor and normal samples for BLCA, n = 112 for BRCA, n = 9 for CHOL, n = 41 for COAD, n = 43 for HNSC, n = 72 for KIRC, n = 24 for LIHC, n = 57 for LUAD, n = 49 for LUSC, n = 27 for STAD, n = 23 for UCEC). P, two-sided Student's t test p-value. Each box represents the IQR and median of expression levels in each sample group, whiskers indicate 1.5 times IQR. BLCA: bladder urothelial carcinoma; BRCA: breast invasive carcinoma; CHOL: cholangiocarcinoma; COAD: colon adenocarcinoma; HNSC: head and neck squamous cell carcinoma; KIRC: kidney renal clear cell carcinoma; LIHC: liver hepatocellular carcinoma; LUAD: lung adenocarcinoma; LUSC: lung squamous cell carcinoma; STAD: stomach adenocarcinoma; UCEC: uterine corpus endometrial carcinoma.

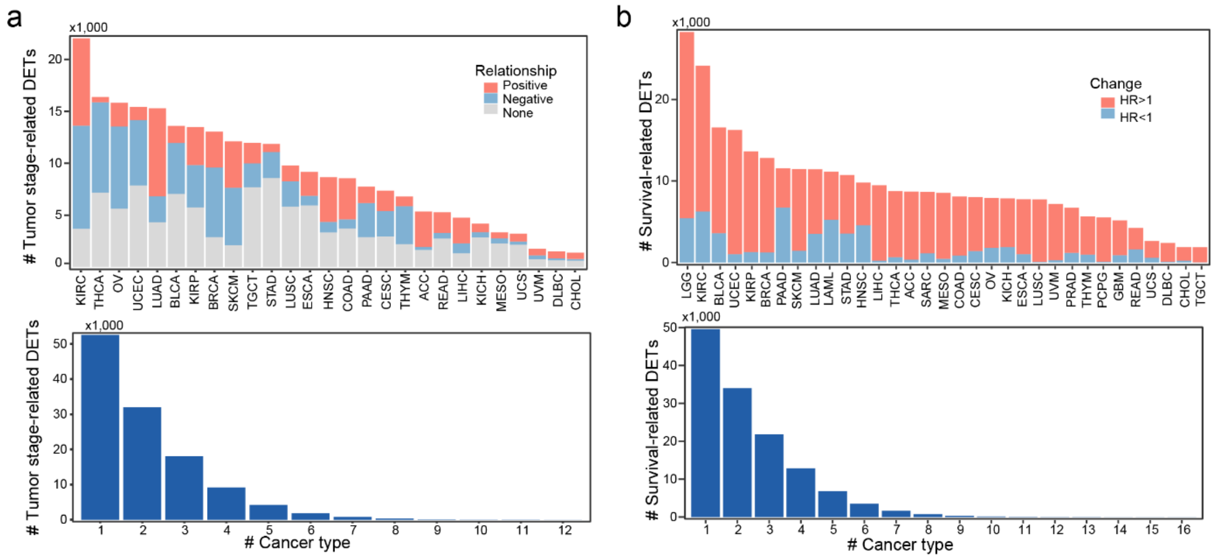

Supplementary Fig. 6 Tumor stage and survival-related unannotated transcripts. a The number of unannotated transcripts that showed differences and association with different tumor stages, and the number of unannotated transcripts that show association with tumor stages in one or multiple cancer types. **b** The number of unannotated transcripts that are associated with patient survival, and the number of unannotated transcripts that show association with patient survival in one or multiple cancer types.

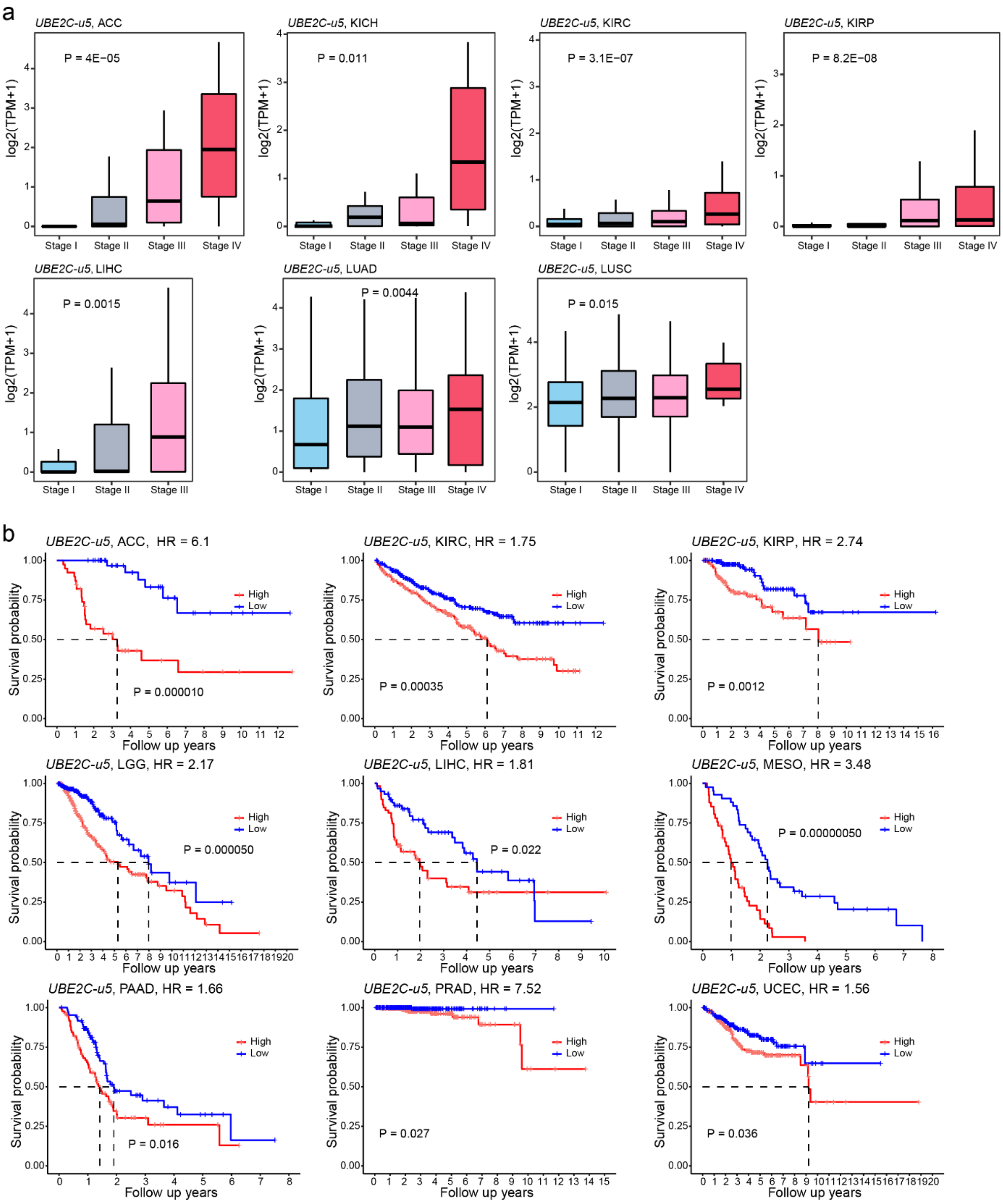

Supplementary Fig. 7 Clinical relevance of *UBE2C-u5* transcript. a Boxplots show comparisons of *UBE2C-u5* transcript among different tumor stages across different cancer types. P, Kruskal-Wallis test p-value. Each box represents the IQR and median of expression levels in each tumor stage, whiskers indicate 1.5 times IQR. n = 9, 37, 16, 15 tumor samples in stage I, II, III, IV for ACC, n = 20, 25, 14, 6 samples for KICH, n = 265, 57, 123, 82 for KIRC, n = 172, 21, 51, 15 for KIRP, n = 44, 28, 42, 2 for LIHC, n = 274, 121, 84, 26 for LUAD, n = 244, 162, 84, 7 for LUSC. **b** Kaplan-Meier survival curves comparing *UBE2C-u5*-high and -low expression groups across different cancer types. P, log-rank test p-value.

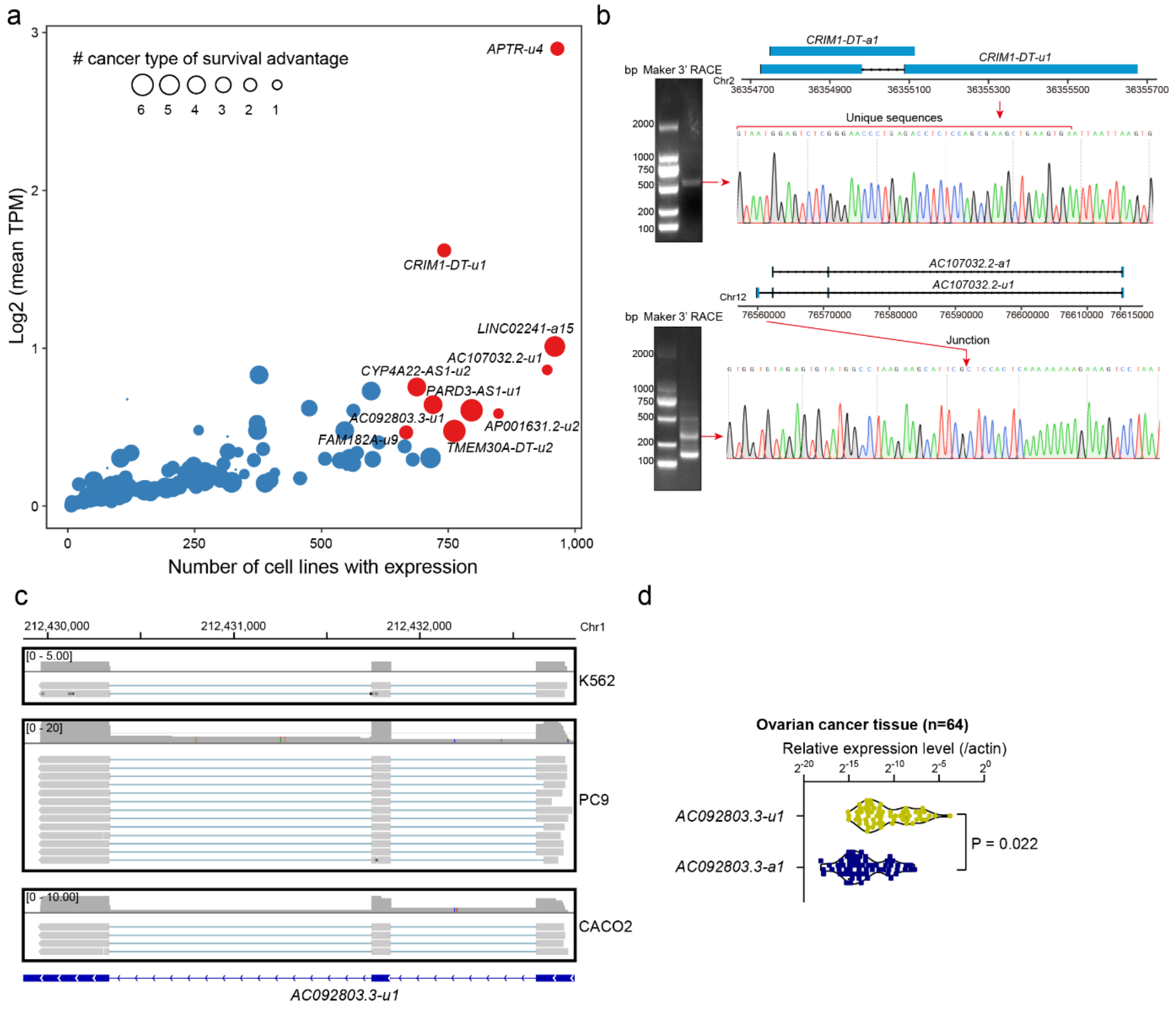

Supplementary Fig. 8 Validation of unannotated lncRNA transcripts. a Scatter plot showing the numbers of cell lines with expression and average expression level of unannotated lncRNA transcripts. The bubble size indicates the number of cancer types that unannotated transcripts have survival significance but the corresponding annotated transcripts don't. **b** Identification of unannotated transcript *CRIM1-D*T-u1 and *AC107032.2-u1* by RACE assay and Sanger sequencing. **c** The *AC092803.3-u1* transcript was overlapped by 2, 13, and 4 long-read RNA sequencing reads in K562, PC9, and CACO2 cell lines, respectively. **d** Comparison of expression levels between *AC092803.3-u1* and *AC092803.3-a1* in 64 ovarian cancer tissue samples. P, two-sided Student's t test p-value.

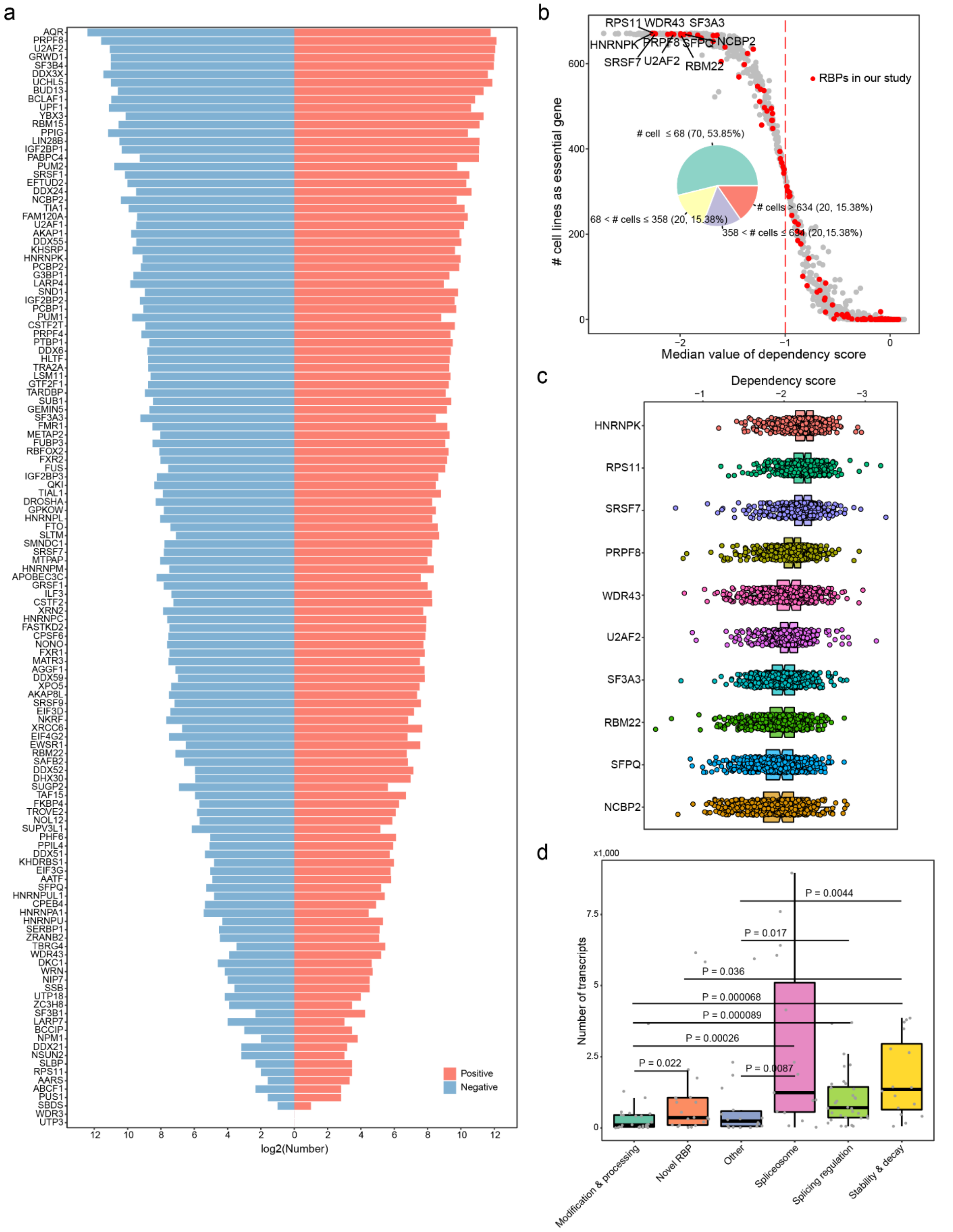

Supplementary Fig. 9 Statistics of RBP-regulated transcripts. a The number of transcripts that are positively or negatively regulated by individual RBPs. **b** The median dependency scores and the numbers of cell lines as essential genes for each RBP gene. Red dots represent those in our study. **c** Box plots showing the distribution of dependency scores of 10 RBPs with the top numbers of cell lines as essential genes and low dependency scores. Each box represents the IQR and median of dependent scores for each RBP, whiskers indicate 1.5 times IQR. n =

671 biologically independent cell lines for each RBP. **d** Boxplots show comparisons of the number of regulated transcripts among different categories of RBPs. P, two-sided Wilcoxon's rank-sum test. Each box represents the IQR and median of transcript numbers for each category, whiskers indicate 1.5 times IQR. n = 27 RBPs for "modification & processing" category, n = 21 RBPs for "novel RBP" category, n = 18 RBPs for "other" category, n = 15 RBPs for "spliceosome" category, n = 32 RBPs for "splicing regulation" category, n = 16 RBPs for "stability & decay" category.

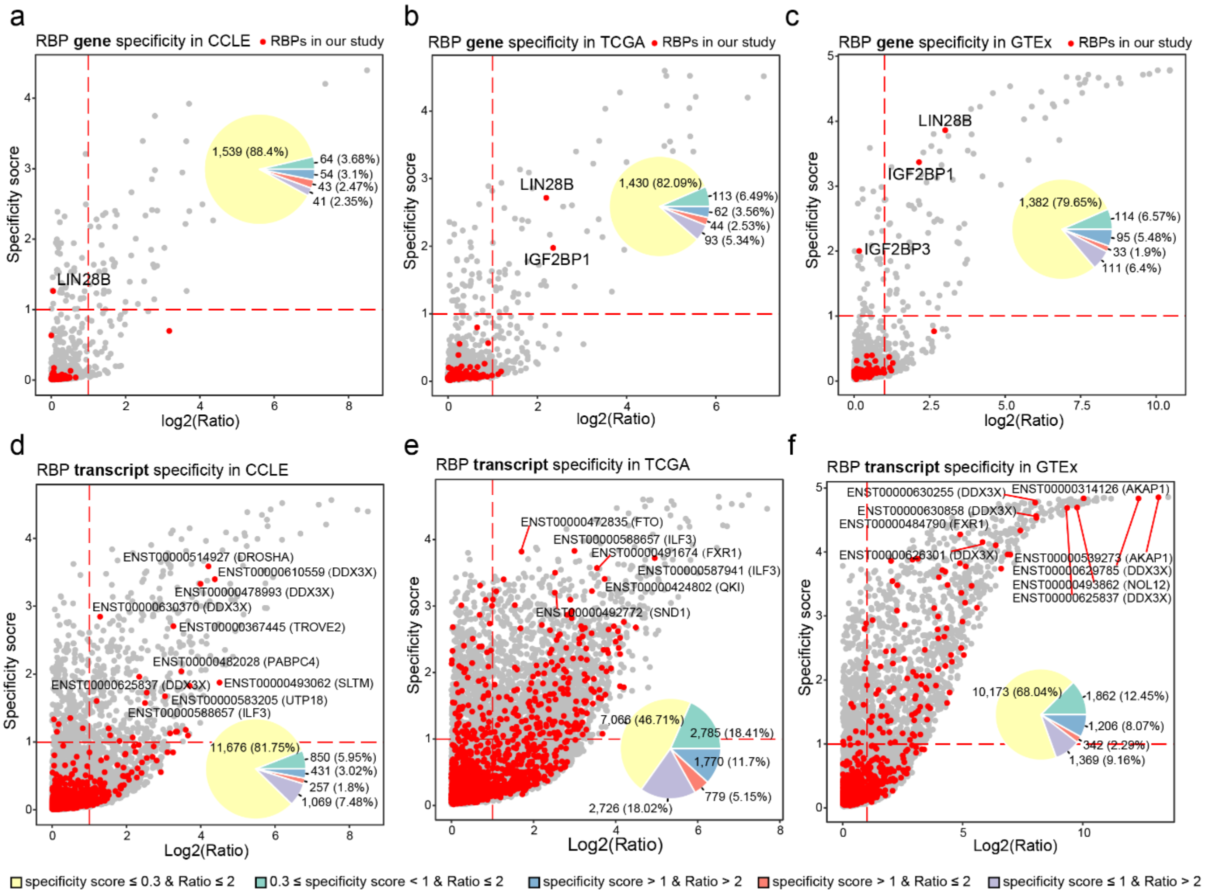

Supplementary Fig. 10 RBP gene and transcript specificity in cancer cell lines (CCLE), primary tumours (TCGA), and normal tissues (GTEx). The specificity scores and ratios of RBP genes in the CCLE (**a**), TCGA (**b**), and GTEx (**c**) datasets. The specificity scores and ratios of RBP transcripts in the CCLE (**d**), TCGA (**e**), and GTEx (**f**) datasets. Red dots represent RBPs in our study. The Ratio indicates the fold change of the highest expression and the second highest expression. The color representation of pie charts was shown at the bottom.

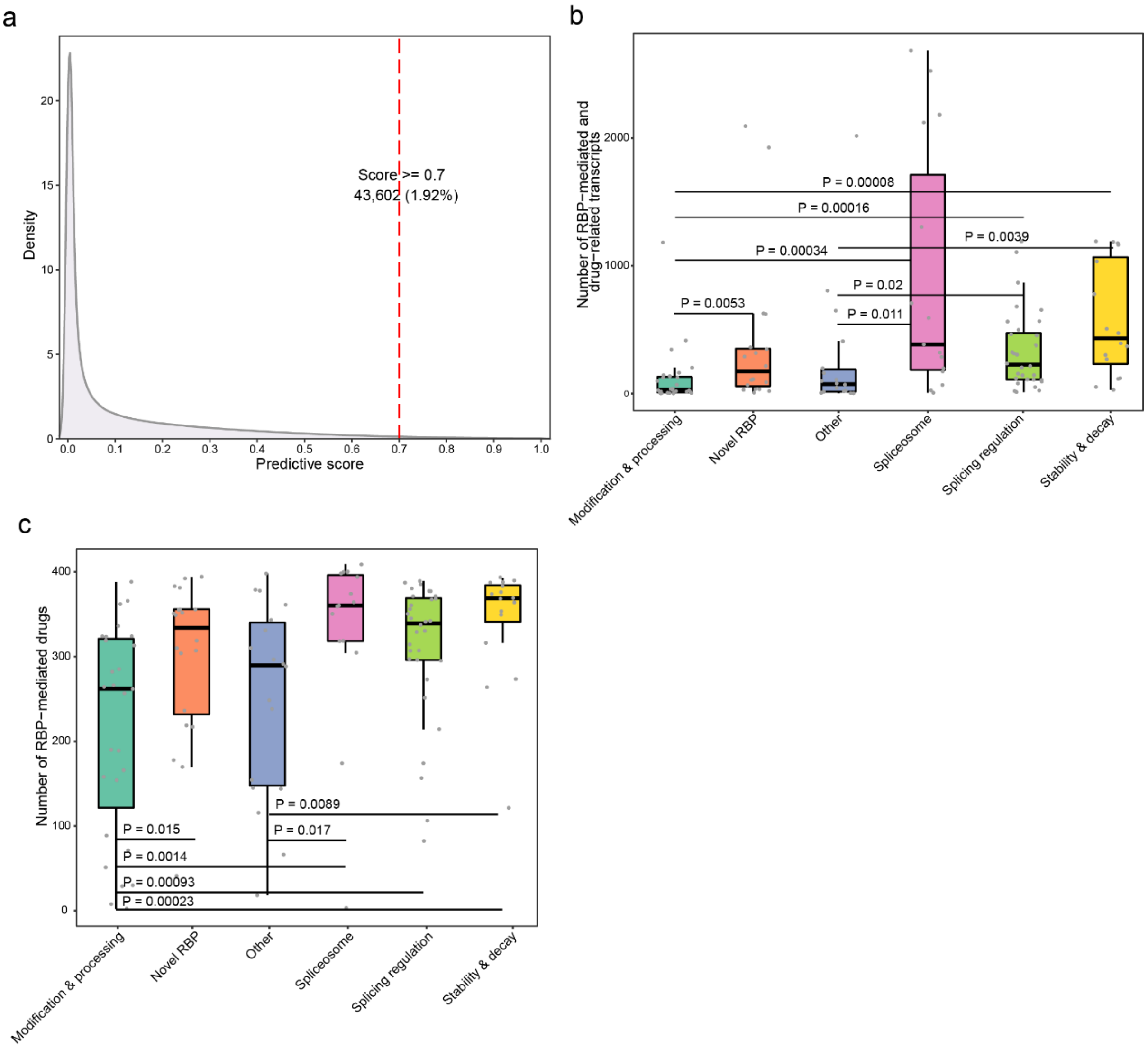

Supplementary Fig. 11 Statistics of transcripts and anti-cancer drugs that are associated with RBPs. a Density distribution curve of predictive scores (> 0) of transcripts for anti-cancer sensitivity. Red vertical line indicates the cutoff for significantly predictive transcripts. **b** Boxplots show comparisons of the number of RBP-regulated drug-associated transcripts among different categories of RBPs. Each box represents the IQR and median of transcript numbers for each category, whiskers indicate 1.5 times IQR. **c** Boxplots show comparisons of RBP-associated anti-cancer drugs among different categories of RBPs. Each box represents the IQR and median of drug numbers for each category, whiskers indicate 1.5 times IQR. In (**b**) and (**c**), two-sided Wilcoxon's rank-sum test was used. n = 27 RBPs for "modification & processing" category, n = 21 RBPs for "novel RBP" category, n = 18 RBPs for "other" category, n = 15 RBPs for "spliceosome" category, n = 32 RBPs for "splicing regulation" category, n = 16 RBPs for "stability & decay" category.

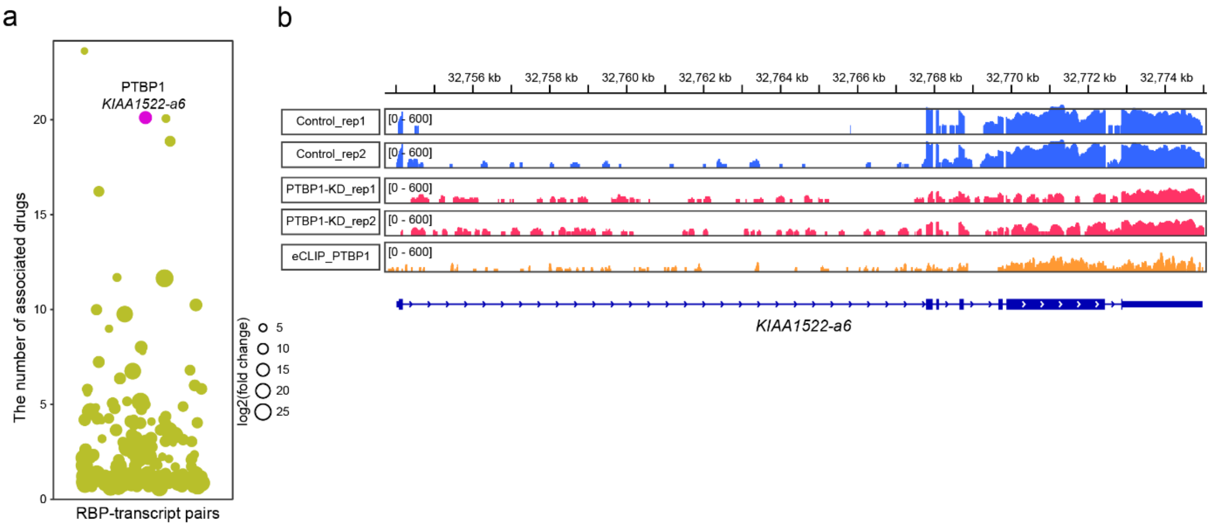

Supplementary Fig. 12 Selection of the *KIAA1522-a6* transcript. a Bubble plot showing the number of associated drugs of RBP-transcript pairs that transcripts were regulated by one certain RBPs. **b** The shRNA-seq and eCLIP-seq signals in *KIAA1522-a6* region.

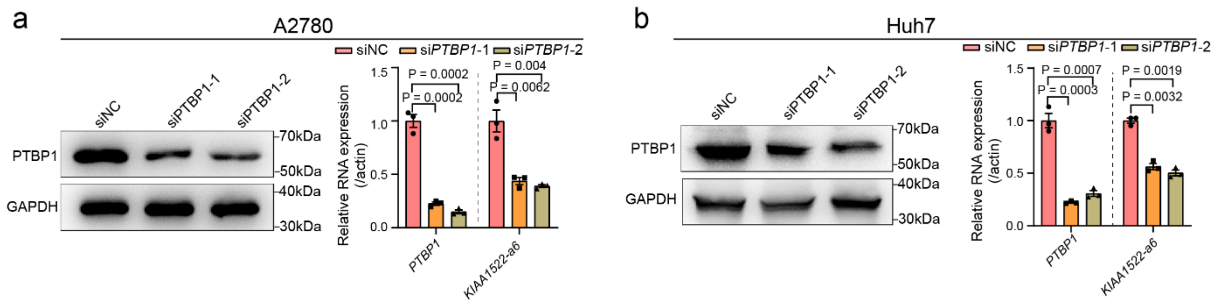

Supplementary Fig. 13 Knock-down effects of different siRNAs targeting *PTBP1*. The relative mRNA and protein expression levels of PTBP1, and the RNA expression level of *KIAA1522-a6* upon siNC, si*PTBP1*-1, and si*PTBP1*-2 in the A2780 cell line (**a**) and the Huh7 cell line (**b**). n = 3 biologically independent samples. Data are presented as mean values +/- SEM.

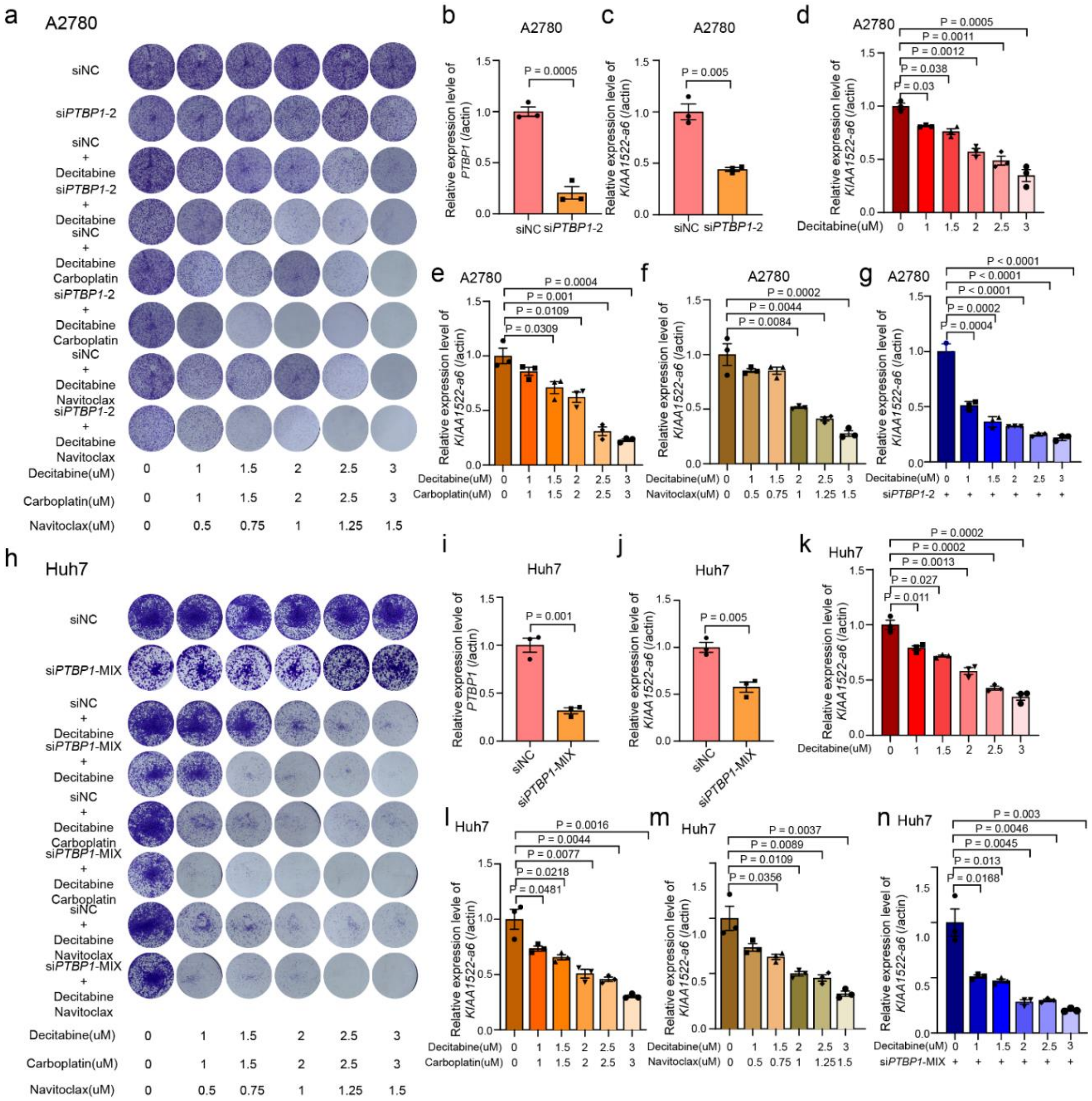

Supplementary Fig. 14 PTBP1-*KIAA1522-a6*-Decitabine axes in cancer cells. a Crystal violet staining of colony formation assays indicates the sensitivity of siNC or si*PTBP1*-2 cells to Decitabine, Decitabine combined with Carboplatin, or Decitabine combined with Navitoclax. Treatment effect is shown for A2780 cells. qRT-PCR assays of the expression of *PTBP1* (**b**) and *KIAA1522-a6* (**c**) after transfection by si*PTBP1*-2 for 48 h in A2780 cells. qRT-PCR assays of the expression of *KIAA1522-a6* in A2780 cells treated with Decitabine (**d**), Decitabine combined with Carboplatin (**e**), and Decitabine combined with Navitoclax (**f**). **g** *KIAA1522-a6* expression in A2780 cells treated with Decitabine combined with si*PTBP1*-2. **h** Crystal violet staining of colony formation assays indicates the sensitivity of siNC or si*PTBP1*-MIX cells to Decitabine, Decitabine combined with Carboplatin, or Decitabine combined with Navitoclax. Treatment effects is shown for Huh7 cells. qRT-PCR assays of the expression of *PTBP1* (**i**) and *KIAA1522-a6* (**j**) after transfection by si*PTBP1*-MIX for 48 h in Huh7 cells. qRT-PCR assays of the expression of *KIAA1522-a6* in Huh7 cells treated with Decitabine (**k**), Decitabine combined with Carboplatin (**l**), and Decitabine combined with Navitoclax (**m**). **n** *KIAA1522-a6* expression in Huh7 cells treated with Decitabine combined with si*PTBP1*-MIX. n = 3 biologically independent samples. Data are presented as mean values +/- SEM.

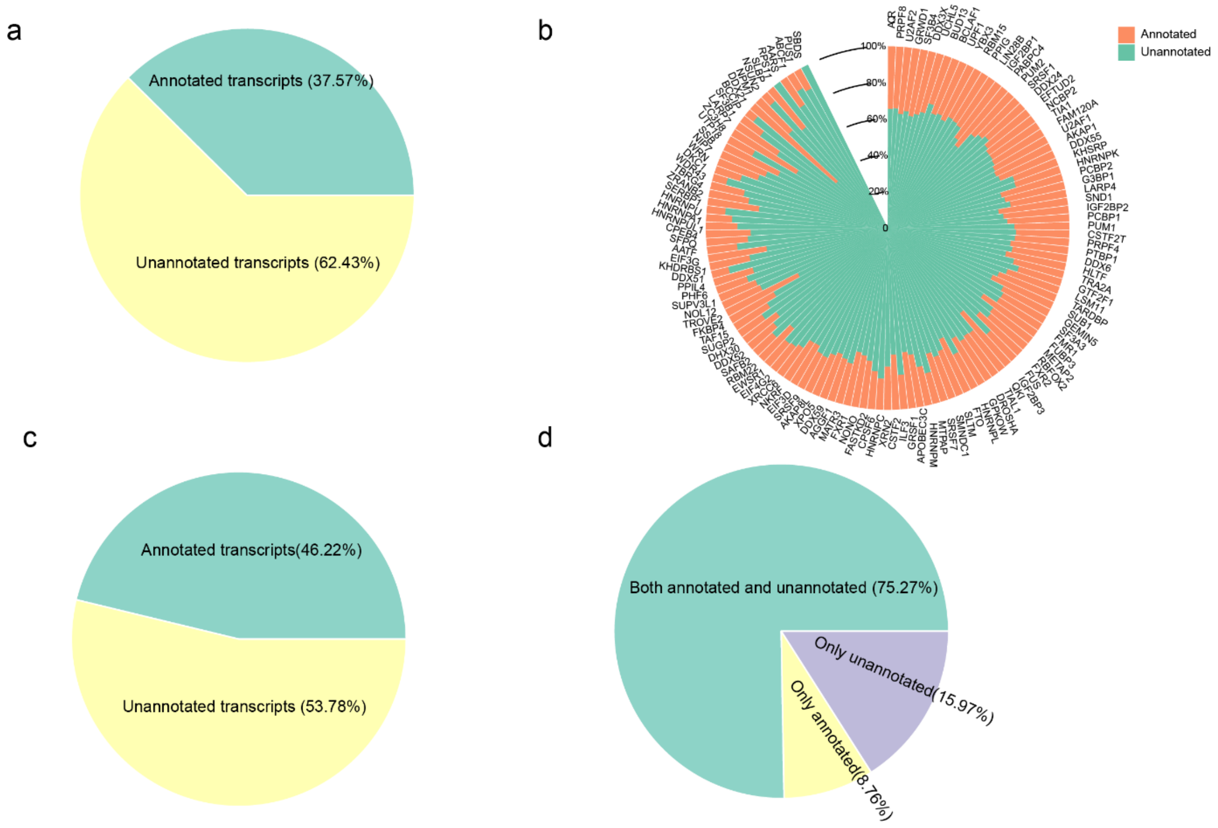

Supplementary Fig. 15 The contribution of unannotated transcripts in RBP and drug networks. a The percentages of annotated and unannotated transcripts in the RBP-transcript regulatory network. **b** The percentages of annotated and unannotated transcripts regulated by each RBP. **c** The percentages of annotated and unannotated transcripts in the RBP-transcript-drug axes. **d** The percentages of RBP-drug connections linked by both unannotated and annotated, only annotated, or only unannotated transcripts.

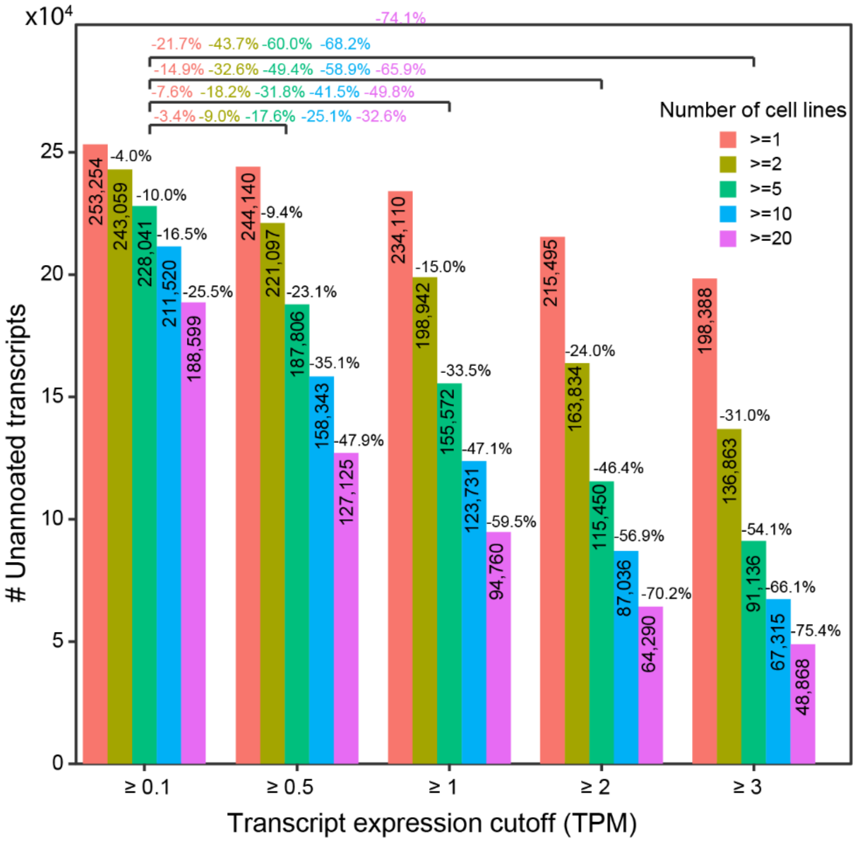

Supplementary Fig. 16 The number of unannotated transcripts when using different cutoff of transcript expression level and sample number.
