# Supplementary material for: Systematic characterization of cancer transcriptome at transcript resolution
Source: Nat Commun. 2022 Nov 10;13:6803. doi: 10.1038/s41467-022-34568-z (PMC9649690; doi:10.1038/s41467-022-34568-z)
Supplement: Supplementary file 2 — Description to Additional Supplementary Information [file 41467_2022_34568_MOESM2_ESM.pdf]

Description of Additional Supplementary Files

Supplementary Data 1. The information of cancer cell lines used in this study.

Supplementary Data 2. The gene type or genomic regions of expressed transcripts.

Supplementary Data 3. Specificity scores and specific tissue types of transcripts.

Supplementary Data 4. The difference of transcripts upon knockdown of different RBPs.

Supplementary Data 5. The expression specificity of RBP genes and transcripts across different human tissues/cancer
