# Supplementary material for: Systematic characterization of cancer transcriptome at transcript resolution
Source: Nat Commun. 2022 Nov 10;13:6803. doi: 10.1038/s41467-022-34568-z (PMC9649690; doi:10.1038/s41467-022-34568-z)
Supplement: Supplementary file 8 — Reporting Summary [file 41467_2022_34568_MOESM8_ESM.pdf]

nature portfolio

Corresponding author(s): Shengli Li

Last updated by author(s): Oct 11, 2022

Reporting Summary

Nature Portfolio wishes to improve the reproducibility of the work that we publish. This form provides structure for consistency and transparency in reporting. For further information on Nature Portfolio policies, see our Editorial Policies and the Editorial Policy Checklist.

Statistics

For all statistical analyses, confirm that the following items are present in the figure legend, table legend, main text, or Methods section.

n/a	Confirmed	
☐	☒	The exact sample size (*n*) for each experimental group/condition, given as a discrete number and unit of measurement
☐	☒	A statement on whether measurements were taken from distinct samples or whether the same sample was measured repeatedly
☐	☒	The statistical test(s) used AND whether they are one- or two-sided<br>*Only common tests should be described solely by name; describe more complex techniques in the Methods section.*
☐	☒	A description of all covariates tested
☐	☒	A description of any assumptions or corrections, such as tests of normality and adjustment for multiple comparisons
☐	☒	A full description of the statistical parameters including central tendency (e.g. means) or other basic estimates (e.g. regression coefficient) AND variation (e.g. standard deviation) or associated estimates of uncertainty (e.g. confidence intervals)
☐	☒	For null hypothesis testing, the test statistic (e.g. *F*, *t*, *r*) with confidence intervals, effect sizes, degrees of freedom and *P* value noted<br>*Give P values as exact values whenever suitable.*
☒	☐	For Bayesian analysis, information on the choice of priors and Markov chain Monte Carlo settings
☒	☐	For hierarchical and complex designs, identification of the appropriate level for tests and full reporting of outcomes
☐	☒	Estimates of effect sizes (e.g. Cohen's *d*, Pearson's *r*), indicating how they were calculated

Our web collection on statistics for biologists contains articles on many of the points above.

Software and code

Policy information about availability of computer code

Data collection	prefetch (version 2.10.8); fasterq-dump (version 2.10.8).
Data analysis	STAR (version 2.7.6a); StringTie (version 2.1.4); Trimmomatic (version 0.39); DESeq2 (version 1.30.0); glment R package (version 4.1); caret R package (version 6.0-86); CPC2 (version 1.01); CPAT (version 3.0.4); survival R package (version 3.4-0); survminer R package (version 0.4.9);FLAIR (version 1.5); minimap2 (version 2.17-r941); GffCompare tool (version 0.12.2); GSVA R package (version 1.38.2); https://github.com/lishenglilab/TAiC/tree/main/Code

For manuscripts utilizing custom algorithms or software that are central to the research but not yet described in published literature, software must be made available to editors and reviewers. We strongly encourage code deposition in a community repository (e.g. GitHub). See the Nature Portfolio guidelines for submitting code & software for further information.

Data

Policy information about availability of data

All manuscripts must include a data availability statement. This statement should provide the following information, where applicable:

- Accession codes, unique identifiers, or web links for publicly available datasets
- A description of any restrictions on data availability
- For clinical datasets or third party data, please ensure that the statement adheres to our policy

The raw RNA-seq data of CCLE project were downloaded from the SRA database (SRP186687, https://www.ncbi.nlm.nih.gov/sra/?term=SRP186687). The eCLIP-seq

and KD-RNA-seq were retrieved from the ENCODE database (https://www.encodeproject.org/). The sensitivity to anti-cancer drugs of cancer cell lines was retrieved from the CTRP database (https://portals.broadinstitute.org/ctrp.v2.1/). The human reference genome and transcript annotation were downloaded from the GENCODE database (https://www.gencodegenes.org/human/release_35.html). Software and resources used for analysis and plotting are described in each method section. All results generated in this study can be found in supplementary tables and the TAiC data portal (http://www.shenglilabs.com/TAiC/).

Human research participants

Policy information about studies involving human research participants and Sex and Gender in Research.

Reporting on sex and gender	Not relevant for this study.
Population characteristics	Not relevant for this study.
Recruitment	Not relevant for this study.
Ethics oversight	Not relevant for this study.

Note that full information on the approval of the study protocol must also be provided in the manuscript.

Field-specific reporting

Please select the one below that is the best fit for your research. If you are not sure, read the appropriate sections before making your selection.

☒ Life sciences ☐ Behavioural & social sciences ☐ Ecological, evolutionary & environmental sciences

For a reference copy of the document with all sections, see nature.com/documents/nr-reporting-summary-flat.pdf

Life sciences study design

All studies must disclose on these points even when the disclosure is negative.

Sample size	For publicly available RNA-seq, eCLIP-seq, KD-RNA-seq, and drug sensitivity data of cancer cell lines, we used all samples in the related projects. In the cancer transcript assembly, we analyzed the raw RNA-seq data of 1,017 cancer cell lines from the CCLE project, which was downloaded from the SRA database (https://www.ncbi.nlm.nih.gov/sra) and listed in Supplementary Data 1. The eCLIP-seq and KD-RNA-seq data of 85 and 107 different RBPs in the HepG2 and K562 cell line, respectively, was retrieved from the ENCODE Project (https://www.encodeproject.org/). Pan-cancer transcript expression analysis was performed in 10,358 samples across 33 cancer types of TCGA, which was downloaded from the GDC data portal (https://portal.gdc.cancer.gov/). In the drug response analysis, we used 481 compounds across 860 cancer cell lines that were retrieved from the Cancer Therapeutics Response Portal (CTRP, https://portals.broadinstitute.org/ctrp/). For the cell growth and drug response experiments, we used 3 biological replicates.
Data exclusions	No data exclusions.
Replication	All data analyses are reproducible. All codes are carefully checked to ensure the replications. Long-read RNA-seq data were analyzed to validate findings in RNA-seq data. Sanger sequencing and PCR were performed to validate findings in RNA-seq data. All validation experiments were replicated 3 times.
Randomization	Cell lines were grouped based on tissue types where they were derived from, and clustered based on correlations and distance. No randomization was involved in this study.
Blinding	The investigators were not blinded as proper controls were already included during experiments design.

Reporting for specific materials, systems and methods

We require information from authors about some types of materials, experimental systems and methods used in many studies. Here, indicate whether each material, system or method listed is relevant to your study. If you are not sure if a list item applies to your research, read the appropriate section before selecting a response.

Materials & experimental systems

n/a	Involved in the study
☐	☒ Antibodies
☐	☒ Eukaryotic cell lines
☒	☐ Palaeontology and archaeology
☒	☐ Animals and other organisms
☒	☐ Clinical data
☒	☐ Dual use research of concern

Methods

n/a	Involved in the study
☒	☐ ChIP-seq
☒	☐ Flow cytometry
☒	☐ MRI-based neuroimaging

Antibodies

Antibodies used	PTBP1 Polyclonal antibody (proteintech,cat#12582-1-AP);GAPDH Monoclonal antibody (proteintech,cat#60004-1-Ig). Various lysates were subjected to SDS PAGE followed by western blot with 12582-1-AP (PTBP1 antibody) at dilution of 1:5000 incubated at room temperature for 1.5 hours. Various lysates were subjected to SDS PAGE followed by western blot with 60004-1-Ig (GAPDH antibody) at dilution of 1:200000 incubated at room temperature for 1.5 hours.
Validation	Antibodies are validated by the vendor. anti-PTBP1, 12582-1-AP, https://www.ptglab.com/products/PTBP1-Antibody-12582-1-AP.htm; anti-GAPDH, 60004-1-Ig, https://www.ptglab.com/products/GAPDH-Antibody-60004-1-Ig.htm.

Eukaryotic cell lines

Policy information about cell lines and Sex and Gender in Research

Cell line source(s)	The A2780 and Huh7 cell lines were purchased from American Type Culture Collection (ATCC).
Authentication	The A2780 and Huh7 cell lines were authenticated by ATCC using Short Tandem Repeat (STR) profiling.
Mycoplasma contamination	Mycoplasma contamination tests in the A2780 and Huh7 cell lines were negative.
Commonly misidentified lines (See ICLAC register)	No cell lines used in this study are commonly misidentified lines in the ICLAC register.
